# Impact of Digital Therapeutics for the Management of Adult Patients With Diabetes: Systematic Review and Meta-Analysis of Randomized Controlled Trials

**DOI:** 10.2196/70428

**Published:** 2025-09-08

**Authors:** Mengyu Li, Ying Chen, Xuehui Chen, Han Yao, Lili You

**Affiliations:** 1 Chinese Academy of Medical Sciences & Peking Union Medical College Beijing China; 2 Hospital for Skin Diseases, Institute of Dermatology, Chinese Academy of Medical Sciences & Peking Union Medical College Nanjing China

**Keywords:** mobile health, mHealth, digital health, digital therapeutics, adult, diabetes mellitus, health management, blood glucose, meta-analysis, systematic review

## Abstract

**Background:**

The growing approval and use of digital therapeutics (DTx) for managing chronic diseases, such as diabetes, has prompted questions about their effectiveness.

**Objective:**

This systematic review and meta-analysis aimed to report the effectiveness of DTx interventions in the management of patients with type 1 diabetes, type 2 diabetes, and prediabetes.

**Methods:**

Data sources, including Web of Science, MEDLINE, Embase, and the Cochrane Library, were searched from inception to July 30, 2023. We included randomized controlled trials assessing the effectiveness of DTx interventions in the health management of individuals with prediabetes and those diagnosed with type 1 diabetes and type 2 diabetes. Our primary outcomes were glycated hemoglobin (HbA_1c_) levels, fasting blood glucose (FBG) levels, BMI, and weight. Trials that met the eligibility criteria were scored for risk of bias using the Cochrane Risk of Bias Tool 2.0. A narrative synthesis and meta-analysis of included studies were also conducted.

**Results:**

A total of 19 studies were included in the meta-analysis (n=3264). The DTx intervention produced a noteworthy reduction in HbA_1c_ levels among individuals with diabetes (weighted mean difference [WMD] −0.54%, 95% CI −0.72 to −0.36), as well as decreases in FBG levels (WMD −0.56, 95% CI −0.76 to −0.37) and BMI (WMD −0.84, 95% CI −1.23 to −0.45). In addition, it contributed to the increases in low-density lipoprotein cholesterol (WMD −0.13, 95% CI −0.22 to −0.03) and triglyceride levels (WMD −0.18, 95% CI −0.34 to −0.02). Subgroup analyses revealed a beneficial impact of DTx on improving HbA_1c_ levels in patients with type 2 diabetes mellitus (mean difference −0.66, 95% CI −0.92 to −0.41). However, this intervention did not elicit a comparable improvement in HbA_1c_ levels for patients with type 1 diabetes mellitus (mean difference −0.45, 95% CI −0.89 to −0.00). DTx did not significantly affect weight reduction (WMD −1.07, 95% CI −2.33 to 0.20), systolic and diastolic blood pressure, total cholesterol levels, or high-density lipoprotein levels.

**Conclusions:**

The DTx intervention holds promise for enhancing health management among individuals with diabetes, potentially attenuating HbA_1c_ levels, FBG levels, and BMI, especially in individuals with type 2 diabetes.

**Trial Registration:**

PROSPERO CRD42023473743; https://www.crd.york.ac.uk/PROSPERO/view/CRD42023473743

## Introduction

Digital therapeutics (DTx) represents a new treatment approach for managing diseases. According to the International Organization for Standardization, DTx is defined as “health software intended to treat or alleviate a disease, disorder, condition, or injury by generating and delivering a medical intervention that has a demonstrable positive therapeutic impact on a patient’s health” [1]. Unlike digital health, which aims to use digital technologies and devices to promote the health of healthy individuals as well as those who are sick, DTx delivers evidence-based therapeutic interventions driven by high-quality software programs for patients. DTx extends treatment to patients’ daily lives and thus offers more opportunities for personalization of interventions compared to traditional medication and lifestyle coaching by physicians in hospitals.

DTx plays an increasingly crucial role in the treatment of diabetes. Currently, an estimated 537 million people are living with diabetes worldwide, resulting in US $966 billion in global health spending, according to the International Diabetes Federation [2,3]. The incidence of this disease is projected to increase to 26.6 million, with a prevalence of 570.9 million, 1.59 million deaths, and 79.3 million disability-adjusted life-years in 2025 without effective interventions [2]. Research shows that the key to controlling diabetes is promoting a healthy lifestyle and modifying risk factors, such as a high BMI, unhealthy diet, and lack of exercise, which DTx can meet perfectly [4,5]. First, DTx coincides with long-term treatment, which the patient with diabetes needs because the apps or wearable devices can be accessible anytime and anywhere [6]. Furthermore, the dynamic and continuous monitoring of DTx enables doctors to gain a more comprehensive understanding of the real-time condition and living environment of patients, thus providing more precise interventions. In addition, DTx drastically reduces the treatment burden on patients and effectively increases compliance by facilitating a convenient operation on the cellphone [7,8]. In addition, compared with visiting the hospital for traditional medical services and medications, DTx is also a cost-saving option [9,10]. Davison et al [11] suggested that a DTx product called BT-001 was cost-effective, with a gain in quality-adjusted life-years of 0.101, saving US $7343 per patient over the lifetime. The US Food and Drug Administration (FDA) has approved several DTx products, such as the Blue Star and Livongo Diabetes Program, as class II medical devices for patients with diabetes. Germany is also in the process of accelerating the development of approval and regulatory rules, and some countries have included DTx in health insurance [12].

However, the development of DTx has encountered some obstacles that need to be solved. Foremost, an increasing number of people with diabetes are using DTx, but limited research and insufficient conclusive evidence are available, leading to mistrust among physicians and patients. The current randomized controlled trials (RCTs) predominantly concentrate on validating the effect of 1 DTx product. In parallel, systematic evidence-based studies have focused primarily on digital health rather than on distinct DTx [11]. In general, the evidence to substantiate the effectiveness of DTx in the management of diabetes is insufficient. Therefore, distinguishing DTx from digital health and telehealth is difficult, which has prevented regulators from determining it as a medical device, thus deferring enrollment for reimbursement. In general, verifying the effectiveness of DTx in improving health quality and reducing the economic burden on patients with diabetes is important. We systematically reviewed the literature on the effectiveness of DTx products for patients with type 1 diabetes, type 2 diabetes, or prediabetes in strict accordance with the concepts prescribed by the Digital Therapy Alliance to address the research gap and determine whether DTx can be evidence based.

## Methods

### Eligibility Criteria

Diagnoses should adhere to the standards that are valid at the time of trial initiation to align with evolving diabetes classification and diagnostic criteria [13]. Ideally, the diagnostic criteria should be described. Where necessary, the authors’ definitions of diabetes mellitus were used. The inclusion criteria were as follows: (1) randomized controlled clinical trials, (2) adults aged ≥18 years with diabetes (prediabetes, type 1 diabetes, or type 2 diabetes), and (3) DTx interventions (the detailed definitions are provided subsequently). The exclusion criteria were as follows: (1) participants aged <18 years (including mixed-age cohorts); (2) DTx was used only for communication between patients and professionals; (3) DTx designed only to provide general education; (4) studies using semiexperimental designs, such as pretest-posttest studies or those lacking control groups; (5) reviews, letters, editorial comments, case reports, conference abstracts, or unpublished articles; and (6) non-English publications.

### Definition of the DTx-Based Intervention

#### Overview

This study establishes eligibility criteria by integrating national regulatory approvals and the definition of DTx by the Digital Therapy Alliance (DTA). If a product has obtained approval in a particular country, it was directly included as an eligible DTx intervention. If it had not undergone country-specific approval, it needed to meet the latest definition of DTx. According to this definition, we consider DTx products to use high-quality software programs to provide personalized interventions for disease or predisease populations, including lifestyle interventions, medication interventions, and comprehensive interventions. From clinical and health economic perspectives, DTx implementation (1) enhances glycemic control through improved self-management behaviors and (2) optimizes health care resource use by shifting chronic disease management to outpatient settings, reducing clinicians’ workload while maintaining intervention efficacy.

#### High-Quality Software Program–Driven Interventions

DTx products should exist as programs, websites, or apps on smartphones or computers. Interventions delivered through media, such as phone calls, SMS text messages, or web-based meetings, were excluded. These products must collect physiological and biochemical indicators, lifestyle indicators, and other relevant data and then use validated algorithms to synthesize, process, and analyze data to generate personalized chronic disease management strategies.

#### Lifestyle Interventions

These interventions involve analyzing and processing patient-related data on movement and dietary structure. Patients can dynamically adjust their carbohydrate and nutrient intake, exercise frequency, and duration based on changes in physiological indicators, such as blood glucose levels and glycated hemoglobin (HbA_1c_) levels, ultimately achieving their ideal weight and optimal dietary habits.

#### Medication Interventions

These interventions primarily involve a continuous analysis and processing of patient indicators related to blood glucose levels, medication habits, and medication dosages. The aim is to dynamically and personally adjust the medication dosage and intervals in real time, standardize and manage the insulin dosage, and improve patients’ medication adherence.

#### Comprehensive Interventions

These interventions refer to combined interventions addressing both patients’ lifestyle and medication use.

### Search Strategy

This meta-analysis followed the PRISMA (Preferred Reporting Items for Systematic Reviews and Meta-analyses) guidelines (Multimedia Appendix 1), and the search was conducted according to the PICO (Population, Intervention, Comparison, and Outcomes) criteria (P: patients with type 1 or type 2 diabetes or prediabetes, I: DTx intervention, C: usual clinical care, and O: diabetes health management effect index). We previously registered the review protocol in the PROSPERO database for systematic reviews (CRD42023473743). Our search strategy was developed through a preliminary review of key papers on DTx [1,6,7] to identify relevant terminology and through consultation with an information specialist from Medical Information of Peking Union Medical College, who assisted in refining search terms and selecting appropriate databases. A total of 4 electronic databases, namely, MEDLINE, the Cochrane Library, Web of Science, and Embase, were searched for global studies. The search strategy was mainly based on the following Medical Subject Headings terms: “(Diabetes Mellitus OR IDDM OR NIDDM OR MODY OR T1DM OR T2DM OR T1D OR T2D OR noninsulin-dependent OR insulin-dependent OR Diabetes Insipidus) AND (Telemedicine OR “digital therapeutic” OR “digital health” OR mHealth OR telemonitoring) AND (randomized controlled trial OR controlled clinical trial OR placebo OR drug therapy OR randomly OR groups).” The search was limited to publications in English from inception to July 30, 2023. For detailed search strategies, please refer to Multimedia Appendix 2. Reference lists of the retrieved articles were also considered when relevant to the issue examined but not allocated in the basic search. Owing to the diverse regulatory frameworks for DTx products across countries, we conducted additional searches following the systematic database review on the FDA website, the German Federal Institute for Drugs and Medical Devices (Bundesinstitut für Arzneimittel und Medizinprodukte), and the product library of the DTA to supplement the inclusion of registered and approved categories of DTx products. We sought the literature supporting the efficacy of these DTx products and incorporated relevant publications that met the inclusion criteria in the analysis. Simultaneously, we retrieved RCTs related to DTx from the US clinical trials registry and supplemented this with clinical trial evidence reported in the literature via PubMed.

### Data Selection and Extraction

The literature screening was conducted using EndNote software (Thomson ResearchSoft). Two reviewers (ML and YC) first removed duplicates from the retrieved articles. In accordance with the inclusion and exclusion criteria and with a clear definition of interventions, they independently screened the abstracts and titles of deduplicated documents. Relevant articles were then selected for a second round of detailed review. The reviewers thoroughly examined the full texts to ensure compliance with the criteria, and eligible studies were included in the qualitative and quantitative syntheses. Disagreements were resolved by a third reviewer (XC) during guidance committee meetings. Information on trial design and data on the primary and secondary outcomes were extracted independently by 2 reviewers using a predesigned Excel spreadsheet (Microsoft).

### Quality Assessment

The risk of bias was assessed using the Cochrane Risk of Bias Tool 2.0 for randomized trials [14]. The following 5 domains were evaluated: bias arising from the randomization process, bias owing to deviations from the intended interventions, bias owing to missing outcome data, bias in the measurement of the outcome, and bias in the selection of the reported result.

A total of 3 to 7 items were used to judge each domain. We classified the risk of bias in each domain as a “low risk,” “high risk,” or “some concerns” and evaluated individual bias items. The overall bias assessment was based on the evaluations across all 5 domains. “Low risk” means that the study was judged to be at a low risk of bias for all domains for that result. “Some concerns” means that the study was judged to raise some concerns in at least 1 domain for that result but was not at a high risk of bias in any domain. “High risk” means that the study was judged to be at a high risk of bias in at least 1 domain for that result or that the study was judged to raise some concerns in multiple domains in a way that substantially reduced confidence in the result.

### Data Analysis

On the basis of critical indicators for a diabetes diagnosis, the perceptibility of variations in indicators owing to the duration of the trial, and the frequency of outcome indicator selection across various RCTs, the primary outcomes can be summarized to encompass HbA_1c_ levels, fasting blood glucose (FBG) levels, weight, and BMI. The remaining secondary indicators were categorized based on intervention effects, encompassing changes in cognition and behavior; indicators related to population health outcomes; and quality of life (QOL)–related indicators, such as adverse events and patient satisfaction. Therefore, indicators related to population health outcomes include systolic blood pressure (SBP), diastolic blood pressure (DBP), postprandial 2-hour blood glucose levels (PBG), total cholesterol levels, high-density lipoprotein cholesterol (HDL-C) levels, low-density lipoprotein cholesterol (LDL-C) levels, triglyceride levels, the percentage change in average body weight, serum creatinine levels, waist circumference, muscle mass, body fat, and the percentage of individuals meeting HbA_1c_ targets. We also included measures of self-management questionnaire scores, adverse event occurrence, and patient satisfaction outcomes. We performed subgroup analyses of the primary findings based on the mean age of the participants, number of participants, intervention mode, intervention duration, study setting, diabetes type, and whether the digital therapeutics software was registered.

For studies meeting the inclusion criteria, 2 reviewers independently extracted data on target populations, interventions, and outcomes using a modified standard template. Discrepancies were resolved through discussion or third-reviewer arbitration. Missing data were sought from the original authors when necessary. Quantitative analyses were performed using Cochrane Review Manager (version 5.4.1) in accordance with the Cochrane guidelines [15]. Continuous outcomes were analyzed by calculating mean differences or average variations where sufficient data existed. For outcomes devoid of means and SDs, categorical data (event frequencies and sample sizes) were used.

We used *I*^2^ statistics and Baujat plots to test heterogeneity. The *I*^2^ statistic, which quantifies inconsistency across studies, was specifically used to assess the impact of heterogeneity on the meta-analysis [16]. Heterogeneity was quantified as low, moderate, or high, with upper limits of 25%, 50%, and 75% for *I*^2^, respectively [17]. When heterogeneity was detected, we identified the potential reasons by scrutinizing individual studies and subgroup characteristics.

A funnel plot and Egger regression analysis (considered significant at *P*≤.10) were used to evaluate the potential presence of small study bias. The interpretation of the funnel plot’s symmetry or asymmetry was used to ascertain the existence of reporting bias, and the potential sources of bias were discussed. We conducted a sensitivity analysis using the single-study knockout approach to assess the contribution of each study to the pooled effect size.

## Results

### Description of Studies

**Figure 1:**
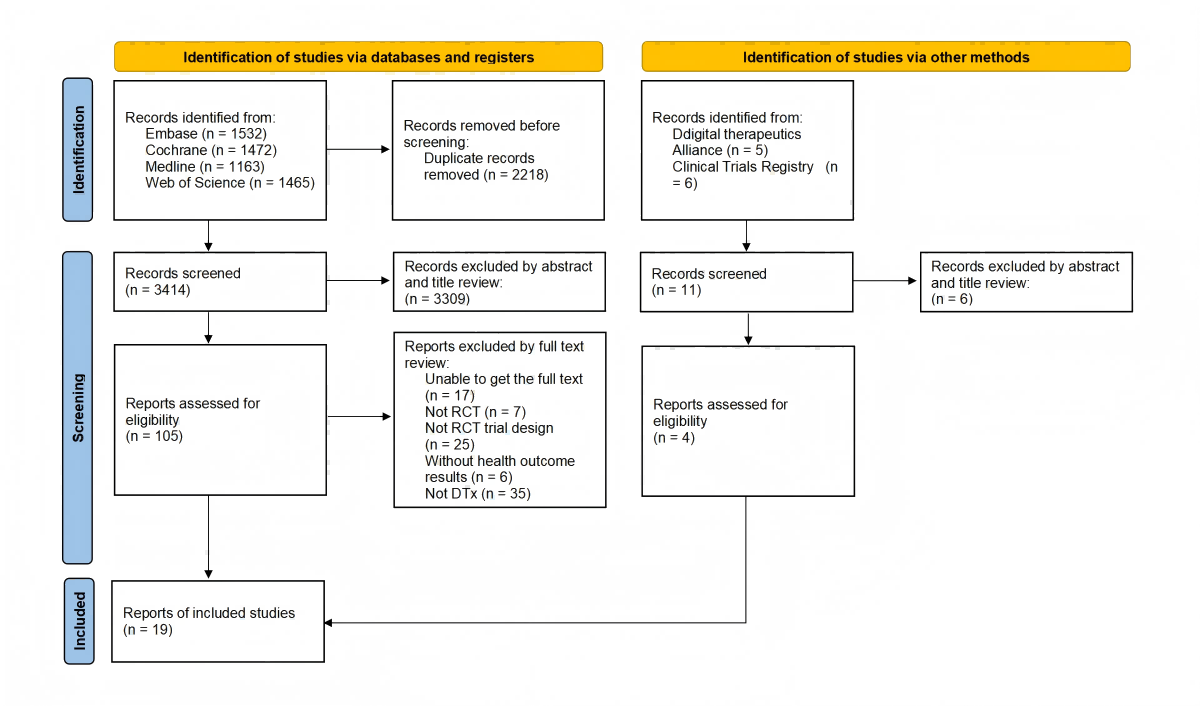
Flow diagram of the study. DTx: digital therapeutics; RCT: randomized controlled trial.

A search of 4 electronic literature databases yielded a total of 5632 abstracts: 1532 (27.2%) from Embase, 1472 (26.1%) from the Cochrane Library, 1163 (20.6%) from MEDLINE, and 1465 (26%) from Web of Science. The flowchart (Figure 1) summarizes this search process. The 2 authors independently assessed 105 full papers for eligibility for inclusion. Fifteen studies, with a total of 2762 participants, met the inclusion criteria and were selected for review. Furthermore, we conducted searches in the product libraries of regulatory authorities, such as the FDA, the German Federal Institute for Drugs and Medical Devices, and the DTA, to identify DTx products that had received regulatory approval. In addition, we retrieved relevant RCTs on DTx from the US ClinicalTrials.gov database. A total of 4 studies involving 502 participants were added through these sources. Ultimately, 19 studies, encompassing 3264 participants, met the inclusion criteria and were selected for comprehensive review and analysis.

In total, 19 studies met the inclusion criteria (Table 1). The characteristics of the included studies are summarized in Multimedia Appendix 3 [18-36]. A total of 7 (37%) studies were conducted in the United States [18-24], 4 (21%) in France [25-28], 1 (5%) in Canada [29], 1 (5%) in Germany [30], 1 (5%) in China [31], 1 (5%) in Indonesia [32], 1 (5%) in South Korea [33], 1 (5%) in Singapore [34], 1 (5%) in the Czech Republic [35], and 1 (5%) in India [36]. All studies were published in English.

**Table 1:** Characteristics of the included studies.

Study	Country	Study design	Population, n	Age (y), mean (SD)	Duration of the intervention	Outcome analysis
Agarwal et al [29], 2019	Canada	RCT^a^	120	IG^b^: 51.5 (10.6); CG^c^: 52.1 (10.7)	6 mo	HbA_1c_^d^, patient-reported diabetes self-care behaviors or general health status, health care use, app use, and satisfaction
Benhamou et al [25], 2019	France	RCT	63	48.2	12 wk	HbA_1c_; the percentage of time spent in the 3.9-10.0 mmol/L glucose target range; the percentage of time with sensor glucose concentration in hypoglycemia or hyperglycemia; coefficient of variation of glucose, low blood glucose index; high blood glucose index; blood glucose risk index; total insulin intake, the number and the amount of carbohydrate intakes; the number of severe hypoglycemic events; the number of severe hyperglycemic episodes or significant ketoacidosis; the number of hypoglycemic episodes; the number of severe hypoglycemic events requiring carbohydrate administration by a third party; the number of severe hypoglycemic events with loss of consciousness; the number of hospital admissions for severe hypoglycemia or ketoacidosis; the number of times carbohydrate was administered and the amount of carbohydrate intake in the last week of each treatment period; the number of technical incidents causing interruptions of the closed loop; the percentage of time spent in the closed-loop functional mode; and assessed patient satisfaction
Bergenstal et al [18], 2019	United States	RCT	181	60.3	6 mo	HbA_1c_, the percentage of HbA_1c_<7% (53 mmol/mol), <8% (64 mmol/mol), and>9% (75 mmol/mol)
Bretschneider et al [30], 2022	Germany	RCT	42	57	3 mo	HbA_1c_, FBG^e^, body weight, and waist circumference
Charpentier et al [26], 2011	France	RCT	163	33.8	6 mo	HbA_1c_, the proportion of patients reaching the HbA_1c_ target of <7.5%, the change in SMPG^f^ frequency, the change in QOL^g^, satisfaction, the amount of time spent by investigators conducting face-to-face visits or teleconsultations, and the amount of time spent by the participants coming for hospital visits
Franc et al [27], 2019	France	RCT	189	58.7	4 mo	HbA_1c_, the percentage of patients reaching HbA_1c_<7.0%, FBG, the percentage of patients reaching FBG73-108 mg/100 mL, pre- and postprandial blood glucose, changes in insulin doses, and QOL
Franc et al [28], 2020	France	RCT	665	38.5	12 mo	HbA_1c_, DIABEO use rates, glucose control improvement, occurrence of hypoglycemia, and QOL
Guo et al [31], 2023	China	RCT	60	IG: 55.25 (13.80); CG: 59.4 (14.59)	—^h^	HbA_1c_, BMI, FBG, 2 h postprandial glucose, QOL, and self-management ability
Hsia et al [19], 2022	United States	RCT	610	58	3 mo	HbA_1c_, FBG, weight, SBP^i^, DBP^j^, total cholesterol, HDL-C^k^, LDL-C^l^, TG^m^, and safety assessments
Hsu et al [20], 2016	United States	RCT	35	53.8	12 wk (−2 wk to +2 wk)	HbA_1c_, the percentage reaching the glycemic target of A1c≤7%, patient satisfaction, the frequency of hypoglycemia, and the time health care professionals and participants spent on managing the insulin titration
Jafar et al [32], 2023	Indonesia	RCT	62	IG: 59.18 (10.24); CG: 54.24 (0.56)	3 mo	HbA_1c_, diabetes self-management knowledge, and QOL
Lee et al [33], 2018	Korea	RCT	136	IG: 51.4 (7.9); CG: 52.6 (7.9)	6 mo	HbA_1c_, BMI, HDL-C, LDL-C, SBP, DBP, SDSCA^n^, and ADS^o^ scores
Lim et al [34], 2021	Singapore	RCT	140	53.1	6 mo	HbA_1c_, weight loss, BMI, FBG, SBP, DBP, total cholesterol, HDL-C, LDL-C, TG, creatinine, years of prediabetes, nutrient intake (calorie, carbohydrate, sugar, protein, total fat, saturated fat, and fiber), physical activity, app use, and proportion of participants with ≥5% weight loss
Moravcová et al [35], 2022	Czech Republic	RCT	100	43.3	6 mo	HbA_1c_, weight reduction, BMI, FBG, waist circumference, muscle mass, body fat, total cholesterol, HDL-C, LDL-C, TG, HOMA-IR^p^, physical fitness, retention, dropout rates, frequencies of interactions, and compliance with the program
Pamungkas et al [36], 2022	India	RCT	60	IG: 56.2 (7.63); CG: 54.5 (9.20)	12 wk	HbA_1c_, SBP, DBP, HDL-C, LDL-C, BMI, and diabetes self-management
Quinn et al [21], 2008	United States	RCT	26	51.05	3 mo	HbA_1c_, SDSCA scores, changes in medication, diet, exercise, patient perception of diabetes management, physician received logbook, new diagnosis depression, diabetes self-care, and self-reported control issues
Sachmechi et al [22], 2023	United States	RCT	78	IG: 58.9 (10.3); CG: 64.5 (13.6)	12 wk	HbA_1c_
Garg et al [23], 2008	United States	RCT	121	IG: 33; CG: 32.5	12 mo	HbA_1c_, body weight, discontinuation, glucose target ranges, hypoglycemia, blood glucose, and insulin dose
Stone et al [24], 2010	United States	RCT	137	—	6 mo	HbA_1c_, weight, SBP, DBP, total cholesterol, HDL-C, LDL-C, TG, SMBG^q^, nurse-to-participant telephone contact time, and medication

^a^RCT: randomized controlled trial.

^b^IG: intervention group.

^c^CG: control group.

^d^HbA_1c_: glycated hemoglobin.

^e^FBG: fasting blood glucose.

^f^SMPG: self-monitoring of plasma glucose.

^g^QOL: quality of life.

^h^Not available.

^i^SBP: systolic blood pressure.

^j^DBP: diastolic blood pressure.

^k^HDL-C: high-density lipoprotein cholesterol.

^l^LDL-C: low-density lipoprotein cholesterol.

^m^TG: triglyceride.

^n^SDSCA: Summary of Diabetes Self-Care Activities.

^o^ADS: Appraisal of Diabetes Scale.

^p^HOMA-IR: Homeostatic Model Assessment of Insulin Resistance.

^q^SMBG: self-monitoring of blood glucose.

In total, 14 (74%) of the 19 studies used routine diabetes care administered by health care professionals as the control group measure, either in isolation or augmented with lifestyle counseling, telephone visits, and glucose monitoring. In 1 (5%) study, a control app was provided in addition to standard care for the control group [19]. Three (16%) studies used a 3-group randomization approach, with 2 (11%) studies allocating standard care to 1 group and assigning the studied app, as well as the app plus telemonitoring, to the other 2 groups [26,28]. In another study [27], standard care, an interactive voice response system, and the studied app were allocated to each of the 3 groups. The remaining 1 (5%) study used a before-and-after self-control design, with the control group consisting of retrospective observations of participants 3 months before using the app [30]. The trial settings varied across studies, with 8 (42%) studies were conducted in hospitals [22,25-29,31,35], 3 (16%) were conducted in specialized diabetes centers [18,20,23], 5 (26%) were conducted in community health centers or community clinics [21,24,32,34,36], 1 (5%) was conducted by an insurance company [33], and 2 (11%) did not report their trial setting [19,30].

The included studies provided results for a total of 3264 participants. The number of participants in individual studies ranged from 30 [21] to 669 [19] (Multimedia Appendix 4 [18-36]). The participants in 11 (58%) of the 19 included studies had type 2 diabetes [18-22,27,29-32,36]; 4 (21%) studies included participants with type 1 diabetes [23,25,26,33]; 1 (5%) study included participants with type 1 or type 2 diabetes [28]; 1 (5%) study included participants with prediabetes [34]; and 1 (5%) study included a mixed population of patients with type 2 diabetes, prediabetes, and insulin resistance, with 67% (67/100) of patients experiencing insulin resistance [35]. The participants in 3 (16%) studies had a mean BMI in the overweight and above range between 29.8 kg/m^2^ [34] and 40.1 kg/m^2^ [35]. The participants in 1 (5%) study were all veterans [24]. The mean age of the participants in most studies ranged from 43.3 [35] to 60.3 years [18]; in 3 (16%) studies, the mean age of the participants ranged from 33.8 to 38.5 years [23,26,28]; and in 1 (5%) study, the participants were aged<80 years, with approximately one-third aged ≥65 years [24] (Multimedia Appendix 5 [18-36]).

The detailed characteristics of each included intervention are outlined in Multimedia Appendix 6 [18-36]. The duration of the intervention varied across studies, ranging from 4 weeks to 12 months: 1 (5%) study lasted 4 weeks [31], 4 (21%) lasted 12 weeks [20,22,25,36], 5 (26%) lasted 3 months [19,21,30,32,35], 1 (5%) lasted 4 months [27], 6 (32%) lasted 6 months [18,24,26,29,33,34], and 2 (11%) lasted 12 months [23,28].

Among the 19 studies included in the analysis, the interventions in 6 (32%) were classified as lifestyle interventions. The apps used during their studies were based on theories, such as the transtheoretical model of behavioral change, the multimodal approach to treatment, and cognitive behavioral therapy, which were combined with procedural algorithms in the form of games, videos, and interactive courses to achieve the personalized generation of intervention strategies while achieving the goals of changing the patient’s lifestyle, improving the patient’s hyperglycemia, and enhancing the patient’s personal self-management skills. The interventions in 7 (37%) studies were pharmacological, and the apps used in the studies all allowed a real-time adjustment of the patient’s insulin injection dosage through built-in algorithms [18,20,23,25-28]. The remaining 6 (32%) studies used a combination of interventions to improve the patients’ glycemic status through various aspects, such as lifestyle and medication use. The apps mainly collected information on and monitored patients’ diet, exercise, and medication compliance by collecting and monitoring their lifestyle information and medical information, such as blood glucose levels and blood pressure, and used algorithms to develop personalized intervention programs. Some also used web-based communication to provide self-management guidance to help patients with diabetes adopt a healthy lifestyle.

Most of the excluded studies pertained to instances wherein the used app was singularly designated for the documentation of routine monitoring data, encompassing metrics such as blood glucose levels and blood pressure. Similarly, excluded studies comprised those focusing exclusively on the archiving of daily lifestyle data, such as dietary habits and physical exercise, or were solely dedicated to diabetes health education. Additional exclusion criteria included investigations involving participants aged<18 years, absence of an RCT design, inquiries featuring solely clinical trial registration particulars without corresponding articles elucidating trial outcomes, and investigations predominantly geared toward the assessment of app safety or usability.

### Risk of Bias in Included Studies

**Figure 2:**
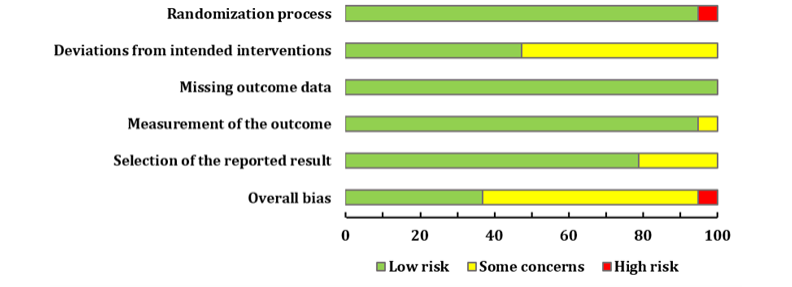
Risk of bias.

**Figure 3:**
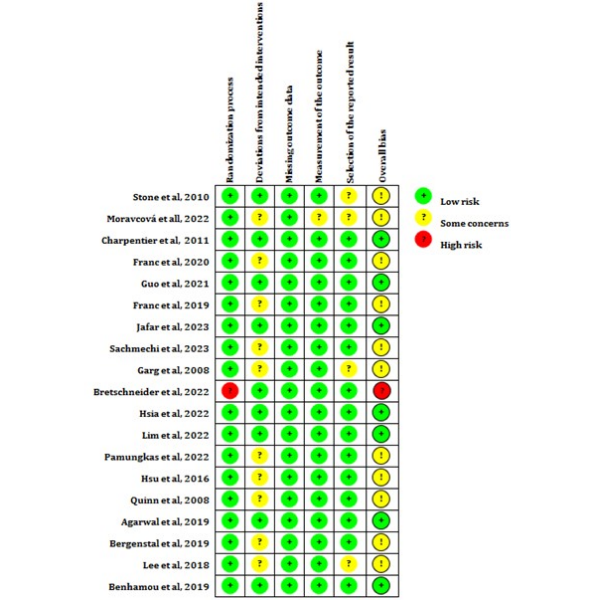
Risk of bias summary.

Among the 19 studies included, 7 (37%) had a low risk of bias, accounting for 36.84%; 11 (58%) had some concerns about the risk of bias, accounting for 57.9%; and 1 (5%) study had a high risk of bias. Among the 11 studies with a risk of “some concerns,” 1 (9%) study [35] was a nonblinded design, and we did not know whether the data analysis was prespecified before the data were collected, while the outcome assessors were probably aware of the intervention previously received by the participants. The participants in 2 (18%) studies [23,33] were aware of their assigned intervention during the trial, and no information about the sequence of data collection and analysis was provided. In 1 (9%) study [24], the concerns of bias related to those in the selection of the reported result because no information was provided. The blinding of physicians and participants was not feasible because the special interventions might have affected the results. Therefore, the remaining 7 (64%) studies were suspected of having a risk of bias because of deviations from the intended interventions. The study by Bretschneider et al [30] was evaluated as high risk because it had a high risk in the domain arising from the randomization process. The study did not report whether the participants were randomly assigned to the intervention group or the control group. A “risk of bias” graph is shown in Figure 2, and a “risk of bias” summary is presented in Figure 3.

### Primary Outcomes

#### HbA1c Levels

**Figure 4:**
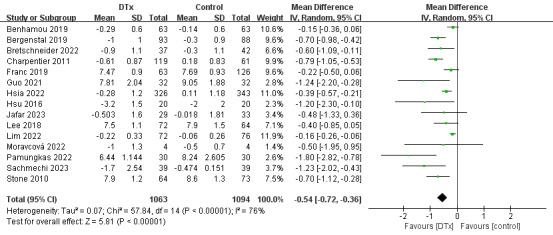
Forest plot showing the effectiveness of digital therapeutics (DTx) for diabetes management in improving glycated hemoglobin levels.

The 19 studies included in the analysis uniformly used HbA_1c_ levels as the outcome measure and reported its variations (Multimedia Appendix 7 [18-36]). Among these, 12 (63%) studies documented statistically significant disparities (*P*<.05) in the changes in HbA_1c_ levels between the intervention and control groups following the cessation of the intervention [18-24,26,30,31,34,36]. In addition, 7 (37%) studies reported reductions in HbA_1c_ levels in the intervention group compared with the baseline level during the study period (*P*<.05) [18-20,24,30,33,35]. Among the 15 (79%) studies assessed, patients with diabetes who received DTx for health management presented a correlation with decreased HbA_1c_ levels (weighted mean difference [WMD] −0.54%, 95% CI −0.72 to −0.36; Figure 4). However, this outcome displayed significant heterogeneity (*I*^2^=76%) that was attributable to the diverse population with diabetes studied, with no correlation observed between patients with type 1 diabetes using DTx for health management and reducing HbA_1c_ levels (WMD −0.45, 95% CI −0.89 to −0.00; Table 2).

**Table 2:** Results from subgroup analysis of the net change in HbA_1c_^a^ and weight.

Subgroup			HbA_1c_									Weight						
			Studies, n		Net change (%, 95% CI)		Heterogeneity				Studies, n			Net change (kg, 95% CI)		Heterogeneity		
							*I*² (%)		*P* value							*I*² (%)	*P* value	
**Duration of the intervention**																		
	<6 mo	8		−0.67 (−1.02 to −0.32)		70		.001		2			3.25 (−1.44 to 7.94)		0			<.001
	≥6 mo	7		−0.51 (−0.74 to −0.27)		83		<.001		6			−1.36 (−2.51 to −0.21)		47			<.001
**Intervention setting**																		
	Hospital	6		−0.57 (−0.94 to −0.21)		78		<.001		2			0.13 (−5.26 to 5.53)		75			<.001
	Diabetes center	2		−0.73 (−1.00 to −0.46)		0		.39		2			−0.79 (−2.23 to 0.65)		0			.99
	Community health center	4		−0.65 (−1.20 to −0.09)		81		.001		2			−1.42 (−6.16 to 3.33)		51			<.001
**Average age**																		
	<55 y	6		−0.41 (−0.69 to −0.13)		79		.<001		3			−0.06 (−1.69 to 1.57)		14			<.001
	≥55 y	8		−0.63 (−0.89 to −0.38)		64		.007		3			−1.99 (−3.42 to −0.56)		50			<.001
**Intervention**																		
	Lifestyle interventions	5		−0.39 (−0.61 to −0.17)		67		.02		4			−1.59 (−3.31 to 0.14)		65			<.001
	Pharmacological interventions	5		−0.51 (−0.83 to −0.19)		56		<.001		3			−0.04 (−2.18 to 2.10)		25			<.001
	Comprehensive interventions	5		−0.94 (−1.46 to −0.41)		76		.06		0			0.00 (0 to 0)		0			<.001
**Disease type**																		
	T1DM^b^	3		−0.45 (−0.89 to −0.00)		86		<.001		1			−0.84 (−2.34 to 0.66)		0			<.001
	T2DM^c^	9		−0.66 (−0.92 to −0.41)		62		.008		3			−0.29 (−1.36 to 0.78)		14			.99
**Number of participants**																		
	<100	8		−0.81 (−1.26 to −0.37)		69		.002		1			−2.30 (−5.03 to 0.43)		31			<.001
	≥100	7		−0.46 (−0.67 to −0.25)		83		<.001		6			−0.84 (−2.33 to 0.59)		49			<.001

^a^HbA_1c_: glycated hemoglobin.

^b^T1DM: type 1 diabetes mellitus.

^c^T2DM: type 2 diabetes mellitus.

#### Proportion of Participants Achieving the Target HbA1c Level

Five (26%) studies examined the proportion of participants who achieved the target HbA_1c_ levels at the end of the study period. Among these, 1 (20%) study [18] reported significant differences between the groups in the proportion of individuals achieving HbA_1c_ levels <7% and <8%. Moreover, 1 (20%) study [34] reported that the likelihood of achieving HbA_1c_ levels <5.7% was 2.1 times higher in the intervention group than in the control group, with a statistically significant odds ratio. Similarly, 1 (20%) study [24] reported that the likelihood of achieving HbA_1c_ levels <7% was 4.4 times higher in the intervention group than in the control group, and the likelihood of achieving HbA_1c_ levels <9% was 2.64 times higher in the intervention group than in the control group, with both odds ratios being statistically significant. However, 2 (40%) studies [23,27] reported no significant differences in the proportions of participants achieving target HbA_1c_ levels between the experimental and control groups.

#### BMI Measures

A total of 7 (37%) studies reported measurements of BMI as an outcome measure [23,30,31,33-36]. Among these, 2 (29%) studies [31,34] documented statistically significant differences in BMI changes between the intervention and control groups, whereas 3 (43%) studies reported a significant reduction in BMI in the intervention group compared with the baseline [23,30,35]. The data from 5 (71%) studies [31,33-36] were adequate to show a significant difference in BMI changes between the intervention and control groups (WMD −0.84, 95% CI −1.23 to −0.45; Multimedia Appendix 8 [18-36]). The integrated intervention had an insignificant effect, whereas the lifestyle intervention had a significant effect. However, a heterogeneity analysis of BMI indicated that studies using lifestyle interventions presented a significant reduction in BMI, whereas those using comprehensive interventions presented no significant changes in BMI (Multimedia Appendix 8).

#### Weight

A total of 9 (47%) studies [18-20,23,24,27,30,34,35] reported weight outcomes. Of these, 1 (11%) study [34] reported statistically significant differences in weight changes between the experimental and control groups, 1 (11%) study [30] reported a significant decrease in weight in the intervention group compared with the baseline at the study’s conclusion, and 3 (33%) studies [18,23,24] reported weight increases in both the intervention and control groups during the study period. The data from 7 (78%) studies [18,19,23,24,27,34,35] were adequate for a meta-analysis (Multimedia Appendix 8), revealing no significant difference in weight changes between the intervention and control groups (WMD −1.07, 95% CI −2.33 to 0.20). However, the heterogeneity analysis of weight indicated that studies with intervention durations >6 months presented a significant decrease in weight levels, whereas those with intervention durations of ≤6 months presented no significant changes in weight levels (Table 2).

#### FBG Levels

A total of 7 (37%) studies [18,19,27,30,31,34,35] reported measurements of FBG levels as an outcome measure. Among these, 4 (57%) studies [18,27,31,34] documented statistically significant differences in changes in FBG levels between the experimental and control groups, whereas 3 (43%) studies [18,30,35] reported significant improvements in FBG levels in the intervention group compared with the baseline level during the study period. The data from 6 (86%) studies [18,19,27,31,34,35] revealed a significant difference in changes in FBG levels between the intervention and control groups (WMD −0.56, 95% CI −0.76 to −0.37; Multimedia Appendix 8).

### Secondary Outcomes

#### SBP and DBP Measurements

A total of 5 (26%) studies [19,24,33,34,36] examined changes in blood pressure following the intervention, with measurements taken for both SBP and DBP. Only 1 (20%) study [36] reported significant differences between the intervention and control groups in both SBP and DBP changes. All 5 (100%) studies were eligible for the meta-analysis (Multimedia Appendix 8), which revealed no statistically significant difference in changes in SBP and DBP between the intervention and control groups after pooling the data.

#### Blood Lipid Levels

A total of 5 (26%) studies [19,24,33-35] reported changes in total cholesterol levels, and after pooling the data for meta-analysis, no significant difference was observed in the changes in total cholesterol levels between the intervention and control groups (WMD −0.11, 95% CI −0.23 to 0.01; Multimedia Appendix 8). Six (32%) studies [19,24,33-36] examined changes in HDL-C and LDL-C levels, with only 1 (17%) study [36] reporting a statistically significant difference in the changes in LDL-C levels between the 2 comparison groups. A meta-analysis of the 6 studies revealed no significant difference in changes in HDL-C levels between the intervention and control groups (WMD 0.09, 95% CI −0.01 to 0.20; Multimedia Appendix 8), whereas a significant difference in changes in the LDL-C levels was observed (WMD −0.13, 95% CI −0.22 to −0.03; Multimedia Appendix 8). Five (26%) studies [19,24,33-35] reported changes in triglyceride levels, with only 1 (20%) study [33] reporting a statistically significant difference in the changes in triglyceride levels between the 2 comparison groups. A meta-analysis incorporating these studies revealed a statistically significant difference in the changes in triglyceride levels between the intervention and control groups (WMD −0.18, 95% CI −0.34 to −0.02; Multimedia Appendix 8).

#### Summary of Diabetes Self-Care Activities Scale

In total, 7 (37%) studies used scales to measure changes in participants’ self-management levels. Only 1 (14%) study [31] reported the use of the Summary of Diabetes Self-Care Activities (SDSCA) scale, revealing that the experimental group scored higher than the control group on the total score and across 5 dimensions, including diet, exercise, blood glucose monitoring, foot care, and smoking, thus demonstrating the effective enhancement of self-management abilities among patients in the experimental group. One (14%) study [32] used the Indonesian version of the Diabetes Knowledge Questionnaire-24 to measure participants’ diabetes self-management (DSM) knowledge. A significant increase in knowledge scores was found in the intervention group after the intervention, with the improvement in the knowledge level of the intervention group surpassing that of the control group, indicating a statistically significant difference in the increase in average scores between the 2 groups. One (14%) study [33] used the SDSCA scale to measure participants’ DSM level and reported improvements in self-management abilities in both groups after the intervention, particularly noting significant differences in the changes in the scores between the group that received the intervention first and the other group, especially for exercise and self-monitoring of blood glucose levels. One (14%) study [36] used a modified version of the DSM questionnaire translated into Indonesian to assess participants’ DSM abilities. The investigation revealed significant improvements in all dimensions of the DSM questionnaire in the experimental group after the intervention, including dietary control, physical exercise, blood glucose monitoring, medication adherence, and screening for diabetes complications, compared with the control group. Finally, 1 (14%) study [21] reported that, compared with the control group receiving standard diabetes care, the experimental group was more likely to have better self-control over diabetes.

The Baujat plot indicated that the studies by Charpentier et al [26] and Lim et al [34] had the most significant impact on overall heterogeneity. On the basis of the funnel plot and Egger test, no significant publication bias was observed in the results. The sensitivity analysis did not reveal any significant changes in the magnitude of the impact of these results (Multimedia Appendix 8).

## Discussion

### Principal Findings

This study is a systematic review of the results of existing RCTs based on a strict definition of digital therapies, aiming to analyze the health effects of digital therapeutic technologies for the treatment and management of patients with diabetes. The primary results showed that core metabolic indicators, including HbA_1c_ levels, FBG levels, BMI, LDL-C levels, and triglyceride levels, improved in patients with diabetes treated with digital therapeutic interventions compared with those in the conventionally treated group, particularly among the type 2 diabetes population. Notably, the effect of digital therapeutics on patients’ weight loss increased progressively with the duration of the intervention, suggesting that cumulative benefits can be generated through sustained digital therapeutics interventions. However, the study revealed that DTx interventions did not significantly impact weight, SBP, DBP, total cholesterol levels, or HDL-C levels in pooled analyses.

### Strengths and Limitations

First, the heterogeneity of included studies poses a key limitation. Despite subanalyses stratified by intervention duration and software features to mitigate this, residual heterogeneity in RCT designs, particularly variations in control groups (usual care vs active comparators), may have inflated variance estimates and reduced statistical power. Second, methodological inconsistencies in HbA_1c_ measurement protocols across studies introduced measurement bias risks, though we prioritized studies using standardized diagnostic criteria to partially offset this. Third, this is a rapidly evolving field, and therefore, the relevance of older studies may be questionable, and new studies may have been published since the search was conducted. We recommend annual updates to capture emerging DTx platforms. Fourth, the lack of standardized digital health terminology during database searches could have omitted pertinent studies, although we used telemedicine to maximize the search range. Future research should adopt CONSORT (Consolidated Standards of Reporting Trials)–Digital Health extensions to improve protocol harmonization and outcome reporting consistency.

Despite the aforementioned limitations, to the best of our knowledge, this study is the first to address the existing gap in the literature regarding the effectiveness of DTx interventions for health management in patients with diabetes. By synthesizing evidence from 19 studies on the efficacy of DTx in improving diabetes health management, our work provides a more comprehensive body of evidence regarding both article quantity and research innovation than previous reviews. Furthermore, rigorous methods were used, following the strict criteria outlined in the PRISMA and Cochrane guidelines, involving a series of comprehensive subgroup analyses and sensitivity analyses to ensure the robustness of the calculated overall effect size. In addition, our study also benefited from the exploration of other outcome measures, including various physiological and biochemical end points, as well as patient-reported indicators, such as patient satisfaction [37]. Besides, our search was limited to publications in English, which may have introduced language bias and excluded relevant studies published in other languages. While we mitigated this through iterative keyword testing with a medical librarian to maximize coverage, this restriction could reduce the geographic representativeness of findings, particularly for region-specific digital health adoption patterns. Future reviews should incorporate multilingual databases (eg, Latin American and Caribbean Literature in Health Sciences and China National Knowledge Infrastructure) to minimize cultural bias.

### Comparison With Prior Work

A considerable body of research is available both domestically and internationally on the feasibility and therapeutic effects of digital health, telemedicine, remote wearable devices, and telehealth, which have been widely applied in various diseases, such as cardiovascular disorders, neurological conditions, chronic pain, and autism, among others [38,39]. However, in the emerging field of digital therapeutics, which is characterized by strict definitions, the current research tends to focus on comprehensive reviews detailing the definition of digital therapeutics [40,41] and elucidating its clinical efficacy in patients with various diseases through RCTs of different products [42,43]. Several RCTs have indicated that digital therapeutic products can enhance the subsequent health management outcomes of patients with diabetes [18-20]. Nevertheless, comprehensive studies on the overall effectiveness of digital therapeutics in health management for patients with diabetes are relatively scarce, and the definition lacks strict uniformity.

Summarizing results from previous studies on the broad concept of mobile health care effectiveness, a study conducted by Agarwal et al [29] also reported significant positive effects of DTx interventions on HbA_1c_ levels. However, DTx interventions did not significantly impact weight, SBP or DBP, total cholesterol levels, or high-density lipoprotein levels, in contrast to several previous studies [44]. This disparity might be attributed to the fact that, on the one hand, patients participating in the study may have been more focused on improvements in their blood glucose levels and related indicators, with less emphasis on comprehensive improvements in diet, exercise, and diabetes knowledge, leading to reduced compliance with other improvement behaviors. On the other hand, lipid control in patients with diabetes relies primarily on the use of medications, and DTx interventions involve precise insulin use and long-term adherence, with less emphasis on drug control interventions for blood pressure and lipid levels, resulting in no significant difference between the two.

In the subgroup analysis of the effects on improving HbA_1c_ levels, a more significant effect was observed in patients with type 2 diabetes, whereas the effect on patients with type 1 diabetes was not significant. The distinct etiologies of type 1 diabetes and type 2 diabetes lead to different treatment emphases. Patients with type 1 diabetes lack insulin; therefore, treatment focuses on insulin infusion therapeutics. In contrast, patients with type 2 diabetes exhibit insulin resistance; thus, treatment not only involves medication but also emphasizes weight and nutrition management through improvements in exercise and diet [45]. Thus, the varied applicability and suitability of DTx products for patients with different types of conditions may result in suboptimal intervention efficacy. In addition, patients with type 1 diabetes, who are generally younger than patients with type 2 diabetes, may have greater acceptance of emerging technologies and greater proficiency in using electronic information technology. Therefore, we recommend designing software with a patient-centric approach for self-monitoring and personalized feedback to enhance user engagement and compliance. Tailoring app functionalities to users based on age, sex, diabetes type, and geographic location [46] is advised. Considering the convenience for the older adult population, designing versions for patients of different ages or simplifying the software information upload process and enlarging software page layouts can facilitate readability and usability for older adults, thus enhancing the specificity and effectiveness of mobile therapeutic interventions.

A subgroup analysis based on intervention duration indicated that the impact of digital therapeutics on weight reduction increased over time. However, the influence on diabetes-related outcome indicators, such as HbA_1c_ and FBG control, did not increase over time. A probable cause for this subgroup effect is the association between the effectiveness of DTx interventions and patient compliance. Chronic diseases demand long-term health management, and some DTx interventions exhibit medicinal properties akin to drug treatments, lacking patient friendliness and regular physician follow-up behaviors [47], making the maintenance of long-term compliance challenging. Therefore, future research needs to address the long-term effectiveness of this technology. We recommend that software development companies incorporate patient reward mechanisms or interactive elements into the design of DTx products for patients with diabetes. In addition, health care institutions, when prescribing DTx interventions for patients with diabetes and conducting follow-ups for diabetes health management, should include follow-ups on product use, provide patients with health education on the efficacy of the interventions, and increase patient compliance and enthusiasm for use.

As indicated in the risk of bias summary, most studies (10/19, 53%) had some concerns of bias because of deviations from intended interventions. The DTx products used in the intervention group predominantly relied on software, whereas the control group typically received standard care for diabetes. Achieving complete blinding for participants and investigators is challenging. However, some studies have shown commendable efforts by incorporating certain core functions of DTx into the same software for control groups. The results of the quality assessment suggest that researchers conducting RCTs of DTx should consider presetting the data analysis method and implementing a blinding method for data processors. Furthermore, studies must be rigorously designed by strictly defining the inclusion and exclusion criteria for participants, ensuring that glycemic indexes are comparable at baseline, and providing comprehensive details in the article to ensure the objectivity of RCTs. These measures provide a scientific reference for the application of DTx in diabetes management.

DTx holds transformative potential for global diabetes care, particularly in low-resource settings where access to specialized care is limited. By enabling remote monitoring and personalized feedback, DTx could democratize diabetes management, reduce health care costs, and alleviate burdens on overstretched health systems. However, equitable implementation requires addressing digital literacy disparities, especially among older adults or populations considered socioeconomically disadvantaged. Future DTx designs should prioritize multilingual interfaces, offline functionality, and integration with local health care infrastructures to ensure scalability and cultural relevance.

In addition to clinical outcomes, several studies included in our review documented significant improvements in patient self-management and QOL, which are core benefits of DTx. For example, Agarwal et al [29] reported enhanced diabetes self-care behaviors in the intervention group, as measured by the Problem Areas in Diabetes scale and the SDSCA questionnaire. Similarly, Charpentier et al [26] observed improvements in QOL among patients using digital interventions, as assessed by the Diabetes Health Profile and Diabetes QOL questionnaires. These findings highlight the unique person-centered impact of DTx, which extends beyond traditional clinical metrics and contributes to better patient engagement and overall well-being.

### Conclusions

The studies reported here describe functional and acceptable technology-based approaches, in addition to conventional care, to increase weight loss in patients with diabetes through the promotion of a healthy lifestyle and improvement in users’ well-being. However, the large heterogeneity in study designs, settings, intervention components, and measurements probably eliminates the strength of this conclusion. This field is evolving so rapidly that the technology used in studies is often outdated by the time results are published. Therefore, similar meta-analytic approaches should be repeated on a regular basis. In addition, patient satisfaction with the DTx can be very informative regarding the design and use of the solution and can provide insights for developers. Future efforts should focus on the personalized development and formulation of interventions; long-term effectiveness studies; and enhancing the specificity, comprehensiveness, and effectiveness of DTx. In addition, our study highlights a limited number of cost-effectiveness analyses for DTx. We recommend that future research expand the exploration of DTx in health economics, improve economic benefit analyses, and provide a foundation for considering the inclusion of DTx in health insurance and its widespread implementation in grassroots health care institutions.

## Appendix

Multimedia Appendix 1 PRISMA checklist.

Multimedia Appendix 2 Search strategy.

Multimedia Appendix 3 Characteristics of studies.

Multimedia Appendix 4 Overview of study populaions.

Multimedia Appendix 5 Baseline characteristics.

Multimedia Appendix 6 Description of interventions.

Multimedia Appendix 7 Original study data.

Multimedia Appendix 8 Result charts.

## Abbreviations

CONSORT: Consolidated Standards of Reporting Trials
DBP: diastolic blood pressure
DSM: diabetes self-management
DTA: Digital Therapy Alliance
DTx: digital therapeutics
FBG: fasting blood glucose
FDA: Food and Drug Administration
HbA1c: glycated hemoglobin
HDL-C: high-density lipoprotein cholesterol
LDL-C: low-density lipoprotein cholesterol
OR: odds ratio
PICO: Population, Intervention, Comparison, and Outcomes
PRISMA: Preferred Reporting Items for Systematic Reviews and Meta-Analyses
QOL: quality of life
RCT: randomized controlled trial
SBP: systolic blood pressure
SDSCA: Summary of Diabetes Self-Care Activities
WMD: weighted mean difference

## Data Availability

Data is supplied in supporting files available for download along with the published manuscript.
