# Supplementary material for: Impact of Digital Therapeutics for the Management of Adult Patients With Diabetes: Systematic Review and Meta-Analysis of Randomized Controlled Trials
Source: J Med Internet Res. 2025 Sep 8;27:e70428. doi: 10.2196/70428 (PMC12455173; doi:10.2196/70428)
Supplement: Multimedia Appendix 2 [file jmir_v27i1e70428_app2.pdf]

MEDLINE

1. exp Diabetes Mellitus/
2. diabet$.tw,ot.
3. (IDDM or NIDDM or MODY or T1DM or T2DM or T1D or T2D).tw,ot.
4. (non insulin$ depend$ or noninsulin$ depend$ or non insulin?depend$ or noninsulin?depend$).tw,ot.
5. (insulin$ depend$ or insulin?depend$).tw,ot.
6. exp Diabetes Insipidus/
7. diabet$ insipidus.tw,ot.
8. 1 or 2 or 3 or 4 or 5
9. 6 or 7
10. 8 not 9
11. exp Telemedicine/
12. ("digital therapeutic*" or "digital health*" or mhealth* or telemonitoring*).tw,ot.
13.11 or 12
14. randomised controlled trial.pt.
15. controlled clinical trial.pt.
16. randomi?ed.ab.
17. placebo.ab.
18. drug therapy.fs.
19. randomly.ab.
20. trial.ab.
21. groups.ab.
22. 14 or 15 or 16 or 17 or 18 or 19 or 20 or 21
23. 10 and 13 and 22

EMBASE search strategy

#1. 'diabetes mellitus'/exp

#2. ‘diabet$’:ab,kw,ti or ‘non insulin* depend*’:ab,kw,ti or ‘noninsulin* depend*’:ab,kw,ti or ‘non insulin?depend*’:ab,kw,ti or ‘noninsulin?depend*’:ab,kw,ti or ‘insulin* depend*’:ab,kw,ti or ‘insulin?depend*’:ab,kw,ti or ‘IDDM’:ab,kw,ti or ‘NIDDM’:ab,kw,ti or ‘MODY’:ab,kw,ti or ‘T1DM’:ab,kw,ti or ‘T2DM’:ab,kw,ti or ‘T1D’:ab,kw,ti or ‘T2D’:ab,kw,ti

#3. #1 or #2

#4. ‘Diabetes Insipidus’/exp

#5. ‘diabet* insipidus’:ab,kw,ti

#6. #4 or #5

#7. #3 not #6

#8.' Telemedicine '/exp

#9.’digital therapeutic*’:ab,kw,ti or ‘digital health*’:ab,kw,ti or ‘mhealth*’:ab,kw,ti or ‘telemonitoring*’:ab,kw,ti

#10. #8 or #9

#11‘Randomized Controlled Trial’/exp

#12.'crossover procedure':de OR 'double-blind procedure':de OR 'randomized controlled trial':de OR 'single-blind procedure':de OR (random* OR factorial* OR crossover* OR cross NEXT/1 over*

OR placebo* OR doubl* NEAR/1 blind* OR singl* NEAR/1 blind* OR assign* OR allocat* OR volunteer*):de,ab,ti
#13.#11 or #12
#14.#7 and #10 and #13

The Cochrane Library
#1 MeSH descriptor: [Diabetes Mellitus] explode all trees
#2 (diabet*):ti,ab,kw or (IDDM):ti,ab,kw or (NIDDM):ti,ab,kw or (MODY):ti,ab,kw or (T1DM):ti,ab,kw or (T2DM):ti,ab,kw or (T1D):ti,ab,kw or (T2D):ti,ab,kw or (non insulin dependent):ti,ab,kw or (non-insulin depend*):ti,ab,kw or (non-insulin-depend*):ti,ab,kw or (insulin* depend*):ti,ab,kw or (insulin-depend*):ti,ab,kw
#3 #1 or #2
#4 MeSH descriptor: [Diabetes Insipidus] explode all trees
#5 (diabet* insipidus):ti,ab,kw
#6 #4 or #5
#7 #3 not #6
#8.' Telemedicine '/exp
#9.(digital therapeutic*):ti,ab,kw or (digital health*):ti,ab,kw or (mhealth*):ti,ab,kw or (telemonitoring*):ti,ab,kw
#10. #8 or #9
#11. MeSH descriptor: [Randomized Controlled Trial] explode all trees
#12.(randomised controlled trial*):ti,ab,kw or (controlled clinical trial*):ti,ab,kw or (randomised):ti,ab,kw or (placebo*):ti,ab,kw or (drug therapy*):ti,ab,kw or (randomly):ti,ab,kw or (trial*):ti,ab,kw or (groups*):ti,ab,kw
#13. #11 or #12
#14. #7 and #10 and #13

Web - of - Science
#1 TS=(diabet* OR IDDM OR NIDDM OR T1DM OR T2DM OR T1D OR T2D OR insulin* depend* OR non insulin* depend*)
#2 TS=(Telemedicine OR ‘digital therapeutic*’ OR ‘digital health*’ OR mhealth* OR telemonitoring*)
#3 TS=(randomized controlled trial* OR controlled clinical trial*OR random* OR clinical trial*)
#4 #1 AND #2 AND #3
