# Supplementary material for: Impact of Digital Therapeutics for the Management of Adult Patients With Diabetes: Systematic Review and Meta-Analysis of Randomized Controlled Trials
Source: J Med Internet Res. 2025 Sep 8;27:e70428. doi: 10.2196/70428 (PMC12455173; doi:10.2196/70428)
Supplement: Multimedia Appendix 3 [file jmir_v27i1e70428_app3.docx]

**Appendix 3. Characteristics of included studies**

**Agarwal 2019**

| Methods | **Study design**: A multicenter, pragmatic randomized controlled trial with blinded outcome assessment designed to evaluate the effectiveness of the BlueStar app |
| --- | --- |
| Participants | **Inclusion criteria**:  1. Adults aged older than 18 years  2. Obtaining care for T2DM at a participating DEP  3. HbA1c ≥ 8.0% (and at least 1% above the participant’s target level) on most recent laboratory report within the last 3 months  4. Currently using an active email address or able and willing to obtain one  5. Able to read the English language (self-reported). **Exclusion criteria**:  1. Have type 1 diabetes  2. Were on continuous glucose monitoring  3. Had an insulin pump  4. Were on dialysis  5. Pregnant  6. Be unable to use a computer or mobile phone because of severe mental or physical impairment |
| Interventions | **Number of centers**: 3  **Country**: Canada  **Setting**: 3 hospital-based diabetes education programs (DEPs) in Ontario, Canada |
| Outcomes | **Primary end point**: HbA1c  **Secondary end points**: patient-reported diabetes self-care behaviors (measured by PAID and Summary of Diabetes Self-Care Activities-6) or general health status (measured by EQ-5D), health care utilization, app utilization, satisfaction |
| Study details | Not terminated before regular end |
| Publication details | **Language**: English  **Funding**: Not mentioned  **Publication status**: Published |
| Stated aim of study | “To conduct a pragmatic randomized controlled trial of the BlueStar mobile app to determine if app usage leads to improved HbA levels among diverse participants in real-life clinical contexts” |
| Bias | There was a low risk of bias. |
| Notes |  |

**Benhamou 2019**

| Methods | **Study design**: A 12-week multicentre, open-label randomised controlled crossover trial |
| --- | --- |
| Participants | **Inclusion criteria**:  1. Patient with type 1 diabetes for at least two years  2. Patient treated with external insulin pump for at least 6 months  3. HbA1c ≤ 10% within the last 4 months  4. Insulin requirements ≤ 50 U per day  5. Patient living in an area covered by a GSM mobile telephone network  6. Non-isolated patient, not living alone, or having a “resource” person living nearby and having a telephone and the key to his/her home  7. Patient not considering a trip out of France during the closed-loop period;8. patient ≥ 18 years old  8. Patient affiliated with social security  9. Patient having signed the free and informed consent form **Exclusion criteria**:  1. Patient presenting any serious pathology that could alter the participation in the study  2. Patient having a long-term treatment with a drug containing paracetamol  3. Patient under legal protection  4. Pregnant patient or patient likely to be pregnant  5. Lack of effective contraception in women of childbearing age;6. breastfeeding patient  6. Psychological and/or physical condition that may affect the proper follow-up of study procedures  7. Severe hypoglycaemia leading to seizures or loss of consciousness in the past 12 months  8. Decreased awareness of hypoglycaemia (Gold score > 4)  9. Impaired renal function (creatinine clearance < 30ml/min)  10. Patient who has had a pancreas or pancreatic islet transplant  11. Patient with serious and not corrected hearing and/or visual problems  12. Patient refusing to perform two capillary blood glucose measurements daily |
| Interventions | **Number of centers**: 12  **Country**: France  **Setting**: 12 university hospitals in France |
| Outcomes | the percentage of time spent in the 3.9 ~ 10.0 mmol/L (70 ~ 180 mg/dL) glucose target range based on continuous glucose monitoring during the 12-week treatment period, the percentage of time sensor glucose concentration, time with glucose concentrations in hyperglycaemia，HbA1c，coefficient of variation of glucose, low and high blood glucose index and blood glucose risk index, total insulin intake, the number and the amount of carbohydrate intakes, the number of severe hypoglycaemic events, the number of severe hyperglycaemic episodes or significant ketoacidosis, number of hypoglycaemic episodes, number of severe hypoglycaemic events requiring intervention of a third party for sugaring, number of severe hypoglycaemic events with loss of consciousness, number of hospital admissions for severe hypoglycaemia or ketoacidosis, the number of sugarings and amount of carbohydrate intake in the last week of each treatment period, and the number of technical incidents causing interruptions of the closed loop, the percentage of time spent in the closed-loop functional mode and assessed patient satisfaction |
| Study details | Not terminated before regular end |
| Publication details | **Language**: English  **Funding**: Funded by French Innovation Fund, Diabeloop  **Publication status**: Published |
| Stated aim of study | “To assess whether the Diabeloop Generation 1 (DBLG1) hybrid closed-loop artificial pancreas system improved glucose control compared with sensor-assisted pump therapy” |
| Bias | There was a low risk of bias. Eligible participants were randomly assigned (1:1) . |
| Notes |  |

**Bergenstal 2019**

| Methods | **Study design**: A prospective, open-label, multicentre, randomised controlled study |
| --- | --- |
| Participants | **Inclusion criteria**:  1. Aged 21 ~ 70 years old at screening  2. Diagnosed with type 2 diabetes with HbA1c of 7.5% or  higher (≥ 58 mmol/mol) and 11% or lower (≤ 97 mmol/mol)  3. Had been using the same insulin regimen for the  previous 3 months  4. With or without other anti-diabetes drugs at a stable dosage for the past 3 months **Exclusion criteria**:  1. Body-mass index (BMI) of 45 kg/m² or more  2. Severe impairment of cardiac, hepatic, or renal functions  3. Psychological or cognitive impairment  4. More than two episodes of severe hypoglycaemic events in the past year  5. A history of hypoglycaemia unawareness  6. A lack of regularly monitored blood glucose |
| Interventions | **Number of centers**: 3  **Country**: USA  **Setting**: three diabetes centers in the USA: the International Diabetes Center at Minneapolis, MN; Henry Ford Medical Center Endocrinology, Detroit, MI; and the Iowa Diabetes and Endocrinology Research Center, Des Moines, IA |
| Outcomes | **Primary end point**: HbA1c  **Secondary end points**: the percentage of HbA1c of less than 7% (53 mmol/mol), less than 8% (64 mmol/mol), and more than 9·0% (75 mmol/mol) |
| Study details | Not terminated before regular end |
| Publication details | **Language**: English  **Funding**: Funded by US National Institutes of Health, National Institute of Digestive and Kidney Diseases  **Publication status**: Published |
| Stated aim of study | “To determine whether the combination of the d-Nav device and health-care professional support is superior to health-care professional support alone” |
| Bias | There were some concerns about risk of bias. It was non-blind to participants and researchers which might cause deviations from the intended interventions. |
| Notes |  |

**Bretschneider 2022**

| Methods | **Study design**: A 3-month, prospective, multicenter, open-label trial with an intraindividual control group |
| --- | --- |
| Participants | **Inclusion criteria**:  1. Over 18 years of age  2. Were able and willing to use Vitadio as part of their diabetes management  3. Baseline HbA1c had to be 6.5~11.0%  4. Enrolled in the disease management program (DMP, a structured treatment program in Germany for patients with chronic diseases) for at least 6 months prior to the study to ensure consistency of care (standard of diabetes care) for the control group **Exclusion criteria**:  1. Using other apps for diabetes management  2. Participating in a weight loss program during prior six months  3. Using a diabetes app in the prior 12 months  4. Treatment with use of an insulin pump or continuous glucose monitoring  5. Impairments which would seriously compromise the integrity of the study—including mental or psychic impairments |
| Interventions | **Country**: Germany  **Setting**: Not mentioned |
| Outcomes | **Primary end point**: HbA1c  **Secondary end points**: fasting glucose, body weight, and waist circumference |
| Study details | Not terminated before regular end |
| Publication details | **Language**: English  **Funding**: Not mentioned  **Publication status**: Published |
| Stated aim of study | “To analyze the extent to which a Digital Flow affects user engagement with a digital platform and impacts activities inherent in diabetes management, such as measuring blood glucose” |
| Bias | There was high risk of bias. Participants were recruited via a Facebook campaign targeting all of Germany who were interested. The information in the control group was retrospective and may have high risk in domain of arising from the randomization process. |
| Notes |  |

**Charpentier 2011**

| Methods | **Study design**: A 6-month, randomized, open-label, parallel-group, multicenter trial |
| --- | --- |
| Participants | **Inclusion criteria**:  1. Over 18 years old  2. Had type 1 diabetes for at least 1 year  3. Had been treated with a basal bolus insulin regimen for at least 6 months  4. Either with MDI or with a pump  5. Last HbA1c values during the year before and at entry of the study were ≥ 8.0%. **Exclusion criteria**:  Exclusion criteria were participation in a diabetes educational program within 3 months before the study or a clinical condition requiring the patient to receive follow-up more frequently than the quarterly visits scheduled. |
| Interventions | **Number of centers**: 17  **Country**: France  **Setting**: 17 hospital sites in France |
| Outcomes | **Primary end point**: HbA1c  **Secondary end points**: the change in the HbA1c, the proportion of patients reaching the HbA1c target of below 7.5%, the change in SMPG frequency, the change in quality of life (QOL), satisfaction assessed by Diabetes Health Profile and Diabetes QOL questionnaires, the amount of time spent by investigators conducting face-to-face visits or teleconsultations, and by the participants coming for hospital visits |
| Study details | Not terminated before regular end |
| Publication details | **Language**: English  **Funding**: Voluntis provided the Diabeo software, and Orange (Paris, France) provided the smartphone and telephone lines; sanofi-aventis (Bridgewater, NJ) and CERITD funded the study.  **Publication status**: Published |
| Stated aim of study | “To demonstrate that Diabeo software enabling individualized insulin dose adjustments combined with telemedicine support significantly improves HbA1c in poorly controlled type 1 diabetic patients. ” |
| Bias | There was a low risk of bias. Participants were randomly assigned to three groups of equal size and not aware of their assigned intervention during the trial. 162/180 participants’ HbA1c hospital measurement was available for end point analysis. The primary efficacy outcome HbA1c high-performance liquid chromatography assays were performed at baseline and end point on the hospital site. The data were that produced this result analyzed in accordance with a pre-specified analysis plan. |
| Notes |  |

**Franc 2019**

| Methods | **Study design**: A randomized, controlled, open-label 13-month trial |
| --- | --- |
| Participants | **Inclusion criteria**:  1. Had inadequate glycaemic control (HbA1c between 7.5% and 10%)  2. With oral antidiabetics at the maximum tolerated dose (metformin ± sulfonylureas ± dipeptidyl peptidase-4 inhibitors)  3. Required addition of BI.diabetes duration > 3 years  4. Body mass index (BMI) < 40 kg/m^2^ **Exclusion criteria**: Not mentioned |
| Interventions | **Number of centers**: 18  **Country**: France  **Setting**: 18 French hospitals |
| Outcomes | **Primary end point**:  the decrease in HbA1c levels at month 4 (M4)  **Secondary end points**:  1. the percentage of patients reaching HbA1c < 7.0%  2. the percentage of patients reaching FBG between 73 and 108 mg/dL (average value of the last 4 days, measured by a glucometer)  3. FBG values (average of the last 4 days before evaluation)  4. pre- and postprandial BG (8-point profiles)  5. changes in insulin doses  6. quality of life (QOL) (using the Diabetes Health Profile QOL scale as well as items from the Diabetes QOL satisfaction dimension) |
| Study details | Not terminated before regular end |
| Publication details | **Language**: English  **Funding**: Founded by CERITD (a non-profit clinical translational research center located next to Corbeil Hospital)  **Publication status**: Published |
| Stated aim of study | “To evaluate the efficacy and safety of two telemonitoring systems to optimize basal insulin (BI) initiation in subjects with inadequately controlled type 2 diabetes” |
| Bias | There were some concerns about risk of bias. It was non-blind to participants and researchers which might cause deviations from the intended interventions. |
| Notes |  |

**Franc 2020**

| Methods | **Study design**: a 12-month, multicenter, double-randomized, open-label trial |
| --- | --- |
| Participants | **Inclusion criteria**:  1. Adults with type 1 and type 2 diabetes  2. Were poorly controlled with intensive insulin therapy  3. Delivered by multiple daily injections or by continuous subcutaneous insulin injection (two HbA1c values were ≥ 8%; one from less than 3 months and the other of more than 1 month before inclusion) **Exclusion criteria**: Not mentioned |
| Interventions | **Number of centers**: 95  **Country**: France  **Setting**: a nonprofit clinical translational research centre located in Corbeil Hospital, Corbeil-Essonnes, France |
| Outcomes | 1. The reduction in HbA1c levels  2. DIABEO usage rates  3. Redictive factors of both  4. Glucose control improvement and DIABEO use  5. Occurrence of hypoglycemia  6. Quality of life |
| Study details | Not terminated before regular end |
| Publication details | **Language**: English  **Funding**: Sponsored by Sanofi (Gentilly, France)  **Publication status**: Published |
| Stated aim of study | “To investigate the efficacy of DS in conditions close to real-life (TELESAGE study)” |
| Bias | There was a low risk of bias. The eligible patients were randomized 1:1:1 into three arms: arm 1 (standard care), arm 2 (DIABEO® alone) and arm 3 (DIABEO® + telemonitoring delegated by the diabetologists to a nursing staff) so people delivering the interventions and participants may aware of their assigned intervention during the trial. |
| Notes |  |

**Guo 2021**

| Methods | **Study design**: A parallel-group, two-arm randomised controlled pilot trial |
| --- | --- |
| Participants | **Inclusion criteria**:  1. >18 years old and < 75 years old  2. Be awake, alert, and responsive to stimuli  3. No cognitive impairment  4. Be able to complete the questionnaire independently  5. Be able to understand mHealth management methods  6. Be able to operate the mobile app correctly  7. Gave informed consent **Exclusion criteria**:  1. Suffered from severe diabetic complications  2. Had difficulty communicating with others  3. Had any neurological conditions. |
| Interventions | **Country**: China  **Setting**: a general hospital in Hangzhou City |
| Outcomes | BMI, FBG, postprandial two-hour blood glucose (2hPG), HbA1c, quality of life (DSQL) and self-management ability |
| Study details | Not terminated before regular end |
| Publication details | **Language**: English  **Funding**: Funded by Guozhen Health Technology (Beijing) Co., Ltd (grant number (2019)JY102)  **Publication status**: Published |
| Stated aim of study | “To evaluate the effectiveness of mHealth management with an implantable glucose sensor and a mobile application among patients with type 2 diabetes mellitus (T2DM) in China. ” |
| Bias | There was a low risk of bias. Participants were randomly divided into an intervention group and a control group. The results of age, BMI, FBG, 2hPG, HbA1c, quality of life and self-management ability between the two groups before the intervention are shown. The loss of follow-up rate is low Method of measuring the outcome were appropriate. |
| Notes |  |

**Hsia 2022**

| Methods | **Study design**: A randomized, controlled, open-label trial |
| --- | --- |
| Participants | **Inclusion criteria**:  1. Adults with type 2 diabetes  2. With HbA1c 7 to < 11% (53 ~ 97 mmol/mol)  3. Access to a smartphone **Exclusion criteria**:  1. Current smokers  2. Taking prandial insulin or oral corticosteroids  3. With active eating disorders. |
| Interventions | **Country**: USA  **Setting**: Not mentioned |
| Outcomes | **Primary end point**: HbA1c  **Secondary end points**:  Safety assessments included adverse events and adverse device effects, FBG, Weight, SBP, DBP, Total cholesterol, HDL, LDL, Triglycerides |
| Study details | Not terminated before regular end |
| Publication details | **Language**: English  **Funding**: Study funding was provided by Better Therapeutics, Inc. J.H. owns AstraZeneca stock. N.L.G. and M.A.B. are employees of Better Therapeutics, Inc., and own stock in the company.  **Publication status**: Published |
| Stated aim of study | “To evaluate the efficacy and safety of a digital therapeutic application (app) delivering cognitive behavioral therapy (CBT) designed to improve glycemic control in patients with type 2 diabetes” |
| Bias | There was a low risk of bias. |
| Notes |  |

**Hsu 2016**

| Methods | **Study design**: A randomized controlled study |
| --- | --- |
| Participants | **Inclusion criteria**:  Subjects with type 2 diabetes(≥ 18 years of age with HbA1c levels of 9 ~ 14%) who were being started on basal insulin therapy by their treating HCPs. **Exclusion criteria**:  1. With significant visual or hearing impairment  2. Were not proficient in English  3. Were pregnant or lactating  4. Had alcohol dependency  5. Required multiple daily insulin injections were excluded. |
| Interventions | **Country**: USA  **Setting**: a tertiary diabetes center |
| Outcomes | **Primary end point**: HbA1c  **Secondary end points**: the percentage reaching the glycemic target of A1c ≤ 7%, the change between patient satisfaction before and after the study, the frequency for hypoglycemia, and the time HCPs and subjects spent on managing the insulin titration |
| Study details | Not terminated before regular end |
| Publication details | **Language**: English  **Funding**: Not mentioned  **Publication status**: Published |
| Stated aim of study | “To demonstrate that the use of this technique can help individuals who start basal insulin therapy achieve better glycemic control ” |
| Bias | There were some concerns about risk of bias. It was non-blind to participants and researchers which might cause deviations from the intended interventions. |
| Notes |  |

**Jafar 2023**

| Methods | **Study design**: A quasi-experimental non-equivalent control group pretest-posttest design |
| --- | --- |
| Participants | **Inclusion criteria**:  1. Aged 20 to approximately 72 years old  2. With T2DM and an HbA1c level of ≥ 6.5%  3. Be able to use an android smartphone  4. Provided consent to participate **Exclusion criteria**:  1. Live alone  2. Pregnant  3. Had serious medical illness including end stage-renal disease and heart failure  4. Had difficulty performing physical activity |
| Interventions | **Number of centers**: 3  **Country**: Indonesia  **Setting**: Primary care health centers |
| Outcomes | the change in HbA1c levels, diabetes self-management knowledge, and quality of life(DQoL-BCI) |
| Study details | Not terminated before regular end |
| Publication details | **Language**: English  **Funding**: No financial support  **Publication status**: Published |
| Bias | There was a low risk of bias. |
| Stated aim of study | “To identify the effect of Guru Diabetes Apps-Based Health Coaching on the level of glycemic hemoglobin, knowledge, and quality of life in Indonesians, as one of Asian ethnicities. ” |
| Notes |  |

**Lee 2018**

| Methods | **Study design**: A randomized, controlled, open-label study |
| --- | --- |
| Participants | **Inclusion criteria**:  1. Age ≥ 19 years old; smartphone user  2. HbA1c  ≥  6.5% within the last 3 months **Exclusion criteria**:  1. Had serious concomitant disease other than diabetes  2. Malignancy-related histories on admission, myocardial infarction, cerebral infarction or organ transplantation  3. Pregnant or had plans for pregnancy within 6 months  4. Plan to participate in other clinical studies or illiteracy |
| Interventions | **Country**: Korea  **Setting**: Samsung Fire and Marine Insurance (Seoul, South Korea) |
| Outcomes | HbA1c level, the changes in BMI, HDL, LDL, SBP, DBP, SDSCA and ADS scores |
| Study details | Not terminated before regular end |
| Publication details | **Language**: English  **Funding**: Funded by Samsung Fire & Marine Insurance Company  **Publication status**: Published |
| Stated aim of study | “To evaluate the effectiveness, reproducibility, and durability of tailored mobile coaching (TMC) on diabetes management” |
| Bias | There were some concerns bias due to deviations from intended interventions and selection of the reported result. Participants were awarded of their assigned intervention during the trial and no information about sequence of data collection and analysis. |
| Notes |  |

**Lim 2022**

| Methods | **Study design**: A randomized controlled trial |
| --- | --- |
| Participants | **Inclusion criteria**:  1. Aged 21 ~ 75 years old  2. Diagnosed with prediabetes (Prediabetes was defined as impaired fasting glucose of 6.1 ~ 6.9 mmol/L or IGT with 2-h plasma glucose of 7.8 ~ 11.0 mmol/L after a 75-g oral glucose tolerance test.)  3. With a BMI of 23.0 kg/m^2^ or more  4. Owned a smartphone and provided written informed consent  **Exclusion criteria**:  1. Diagnosis of type 1 or type 2 diabetes  2. Diagnosis of heart failure, advanced kidney disease, depression, severe cognitive deficits, untreated hypothyroidism, pregnancy, untreated anemia, known thalassemia or other blood disorders |
| Interventions | **Country**: Singapore  **Setting**: government polyclinics, general practitioner clinics, health screening facilities and hospital outpatient clinics |
| Outcomes | **Primary end point**: weight loss, BMI  **Secondary end points**: HbA1c, FBG, SBP, DBP, Total cholesterol, HDL, LDL, Triglycerides, Creatinine, Years of prediabetes, Nutrient intake(Calorie, Carbohydrate, Sugar, Protein, Total fat, Saturated fat, Fiber), Physical activity, app utilization, Proportion of participants with ≥ 5% weight loss |
| Study details | Not terminated before regular end |
| Publication details | **Language**: English  **Funding**: Funded by the Singapore Ministry of Health’s National Medical Research Council under its Health Services Research Grant (NMRC/HSRG/0063/2016)  **Publication status**: Published |
| Stated aim of study | “To assess whether a smartphone app-based lifestyle intervention program would lead to weight loss, normoglycemia and improved metabolic indices in a multiethnic Asian population with prediabetes” |
| Bias | There was a low risk of bias. |
| Notes | Blinding of participants and investigators was not possible due to the nature of the intervention. |

**Moravcová 2022**

| Methods | **Study design**: A prospective, double-armed, randomized controlled trial |
| --- | --- |
| Participants | **Inclusion criteria**:  Only obese patients (defined as BMI over 30 kg/m^2^) older than 18 years with one of the following conditions were included: diagnosed type 2 diabetes mellitus or prediabetes (defined as fasting glucose within the range of 5.6 ~ 6.9 mmol/L or oral glucose tolerance test (OGTT) within the range of 7.8 ~ 11.0 mmol/L) or insulin resistance (IR) (defined as HOMA-IR > 2.7). **Exclusion criteria**:  1. Insulin therapy or steroid therapy  2. Severe liver or kidney disease  3. Age older than 60 years  4. Pregnancy  5. Inability or unwillingness to use Vitadio or to comply with study procedures |
| Interventions | **Country**: Czech Republic  **Setting**: The Department of Exercise Medicine and Cardiovascular Rehabilitation in the University Hospital Olomouc |
| Outcomes | **Primary end point**: weight reduction  **Secondary end points**: BMI, waist circumference, muscle mass, body fat, total cholesterol, TAG, HDL, LDL, FG, HbA1c , HOMA-IR, ALT, AST, GGT; physical fitness (assessed by spiroergometry) and sleep apnea(not reported); retention, drop-out rates, frequencies of interactions and compliance with the program (such as reading lessons, achieving personal goals, compliance with self-monitoring, etc.) |
| Study details | Not terminated before regular end |
| Publication details | **Language**: English  **Funding**: Received a grant from Palacký University Olomouc (project id: IGA_LF_2020_030)  **Publication status**: Published |
| Stated aim of study | “To evaluate whether the effect of using Vitadio is comparable to that of participating in an intensive individualized weight reduction program administered at a specialized clinic in-person.” |
| Bias | There was risk of bias in 3 domains. Firstly, there are some concerns about deviations from intended interventions because no information indicate whether the non-blind method had an effect on the results. Secondly, there are some concerns about measurement of the outcome because assessors were awarded of the intervention received by participants which might influence the outcome. Thirdly, there was no information whether the data analysis was pre-specified before collecting unblinded data which may cause bias in selection of the reported result. |
| Notes | Blinding of physicians and participants was not feasible due to the nature of the intervention. At the end of the article, a screenshot of the page used by the app is attached. |

**Pamungkas 2022**

| Methods | **Study design**: A randomized controlled trial |
| --- | --- |
| Participants | **Inclusion criteria**:  1. Uncontrolled T2DM patients with HbA1c >7%  2. Aged 35 ~ 59 years old  3. Had been living with DM for more than two consecutive years  4. Be able to communicate in the Indonesian language, both verbal and written  5. Be willingness to participate in this study **Exclusion criteria**:  With serious complications such as foot ulcers, chronic renal diseases, and retinopathy |
| Interventions | **Number of centers**: 3  **Country**: India  **Setting**: three community health centers |
| Outcomes | socio-demographic questionnaire, Indonesian version of diabetes self-management (DSM) questionnaire, HbA1c, SBP, DBP, HDL, LDL, Body mass index |
| Study details | Not terminated before regular end |
| Publication details | **Language**: English  **Funding**: Be granted by Ristekbrin  **Publication status**: Published |
| Stated aim of study | “To examine the effect of a smartphone application of diabetes coaching intervention on improving self-management behaviors and preventing onset diabetes complications” |
| Bias | There were some concerns about risk of bias. It was non-blind to participants and researchers which might cause deviations from the intended interventions. |
| Notes |  |

**Quinn 2008**

| Methods | **Study design**: A nonblinded, randomized controlled trial |
| --- | --- |
| Participants | **Inclusion criteria**:  1. 18 ~ 70 years old who had a diagnosis of type 2 diabetes for at least 6 months  2. Required to have an A1c ≥ 7.5% and to have been on a stable diabetes therapeutic regimen for 3 months **Exclusion criteria**: Not mentioned |
| Interventions | **Country**: USA  **Setting**: one community endocrinology and two community primary care practices |
| Outcomes | SDSCA questionnaire, Changes in medication(Medications intensified, Medication errors identified), A1c, diet, exercise, patient perception of diabetes management, Physician received logbook, New diagnosis depression, Diabetes self-care, Self-reported control issues |
| Study details | Not terminated before regular end |
| Publication details | **Language**: English  **Funding**: Supported by Life Scan, Inc. and Nokia, Inc  **Publication status**: Published |
| Stated aim of study | “The primary study aim was to assess the impact on A1c of a cell phone-based diabetes management software system used with web-based data analytics and therapy optimization tools. Secondary aims examined health care provider (HCP) adherence to prescribing guidelines and assessed HCPs’ adoption of the technology.” |
| Bias | There were some concerns about risk of bias. It was non-blind to participants and researchers which might cause deviations from the intended interventions. |
| Notes |  |

**Sachmechi 2023**

| Methods | **Study design**: A 12-week, prospective, pragmatic, single-center, double-arm study |
| --- | --- |
| Participants | **Inclusion criteria**:  1. Age ≥ 18 years old  2. Diagnosis of type 2 diabetes mellitus, HbA1c level of ≥ 7.5% (58 mmol/mol)  3. Access to a smartphone by the patient or a family member  4. Had the ability to read and write English **Exclusion criteria**:  1. Current treatment with continuous subcutaneous insulin infusion via an insulin pump or chronic steroid use  2. Diagnosis of Cushing syndrome, or chemotherapy |
| Interventions | **Country**: USA  **Setting**: The Diabetes Center of Excellence at Queens Hospital Center in Jamaica, New York |
| Outcomes | **Primary end point**: HbA1c |
| Study details | Not terminated before regular end |
| Publication details | **Language**: English  **Funding**: Not mentioned  **Publication status**: Published |
| Stated aim of study | “To evaluate the effectiveness of the Vivovitals diabetes platform in improving glycemic control and reducing hemoglobin A1c (HbA1c) levels in patients with uncontrolled type 2 diabetes mellitus by providing more accessible and direct patient care under the monitoring and oversight of their physician ” |
| Bias | There were some concerns about risk of bias. It was non-blind to participants and researchers which might cause deviations from the intended interventions. |
| Notes |  |

**Satish 2007**

| Methods | **Study design**: 1-year open label randomized control trial |
| --- | --- |
| Participants | **Inclusion criteria**:  adult subjects with type 1 diabetes with a baseline A1c of 7.5 ~ 11% **Exclusion criteria**:  1. Were pregnant or planning to become pregnant  2. Patients on insulin pumps or taking glucocorticoid therapy  3. Diagnosed with cancer, liver disease, anemia, or hepatitis  4. Exercised more than 5 days a week or often traveled internationally |
| Interventions | **Country**: USA  **Setting**: The Barbara Davis Center for Childhood Diabetes at the University of Colorado at Denver Health Sciences Center (Aurora, CO) |
| Outcomes | Discontinued subjects, HbA1c, Glucose target ranges, Hypoglycemia, Blood glucose, Insulin dose, Body weight |
| Study details | Not terminated before regular end |
| Publication details | **Language**: English  **Funding**: This study was sponsored in part by grant 08 FLA 00250 from the State of Colorado Public Health and Environment; grant P30 DK575616 from the Diabetes Endocrine Research Center, National Institutes of Health; grant M01 RR0069 from the General Clinical Research Centers Program, National Institutes of Health; and grants R01 HL61753, RO1 HL079611, and RO1 DK32493 from the Children’s Diabetes Foundation (Denver, CO).  **Publication status**: Published |
| Stated aim of study | “To see if subjects using insulin dosing advisor software will improve glucose control over 1 year. ” |
| Bias | There were some concerns about risk of bias due to deviations from intended interventions and selection of the reported result. Participants were awarded of their assigned intervention during the trial and no information about sequence of data collection and analysis. |
| Notes |  |

**Stone 2010**

| Methods | **Study design**: Randomized controlled trial |
| --- | --- |
| Participants | **Inclusion criteria**:  1. Had at least one outpatient visit in a primary care clinic between 1 June 2004 and 31 December 2005  2. Aged < 80 years old  3. Received pharmacological treatment for diabetes for ≥ 12 months  4. Had no referrals to the VAPHS Diabetes Clinic in the preceding 18 months  5. Had a most recent A1C ≥ 8.0% **Exclusion criteria**:  1. Had a life expectancy of < 6 months  2. Participating in another study  3. Resided in an institutional setting  4. Did not have a land-based, analog home telephone line as required for the home telemonitoring device used |
| Interventions | **Country**: USA  **Setting**: the VA Pittsburgh Healthcare System (VAPHS) at one of the three main Pittsburgh campuses or five outlying community-based clinics |
| Outcomes | **Primary end point**: HbA1C, SBP, DBP, weight, cholesterol, HDL, LDL, Triglycerides  **Secondary end points**: self-monitored blood glucose (SMBG), Nurse-to-participant telephone contact time, medication |
| Study details | Not terminated before regular end |
| Publication details | **Language**: English  **Funding**: Awarded W81XWH-04-2-0030 from the U.S. Air Force, administered by the U.S. Army Medical Research Acquisition Activity, Fort Detrick, Maryland, and by resources and the use of facilities at the VAPHS  **Publication status**: Published |
| Stated aim of study | “To compare the short-term efficacy of home telemonitoring coupled with active medication management by a nurse practitioner with a monthly care coordination telephone call on glycemic control in veterans with type 2 diabetes and entry A1C ≥ 7.5% ” |
| Bias | There is no information that result analysis in accordance with a pre-specified analysis plan which was finalized before available unblinded outcome data |
| Notes | Because of the nature of the intervention, neither participants nor study nurses could be blinded. However, primary outcomes were ascertained by personnel unconnected to this study who were unaware of intervention assignments. |
