# Supplementary material for: Impact of Digital Therapeutics for the Management of Adult Patients With Diabetes: Systematic Review and Meta-Analysis of Randomized Controlled Trials
Source: J Med Internet Res. 2025 Sep 8;27:e70428. doi: 10.2196/70428 (PMC12455173; doi:10.2196/70428)
Supplement: Multimedia Appendix 4 [file jmir_v27i1e70428_app4.docx]

**Appendix 4. Overview of study populaions**

| Study ID | Characteristic | Value |
| --- | --- | --- |
| Agarwal 2019 | **Intervention(s) and control(s)** |  |
|  | I^a^ | BlueStar (immediate treatment  group, ITG) |
|  | C^b^ | wait-list control group, WLC |
|  | Screened, n | 463 |
|  | **Randomized, n** |  |
|  | I | 110 |
|  | C | 113 |
|  | T^c^ | 223 |
|  | ITT, n |  |
|  | **Finishing study, n** |  |
|  | I | 57 |
|  | C | 63 |
|  | T | 120 |
|  | **Randomized finishing study (%)** |  |
|  | I | 92 |
|  | C | 93 |
|  | T | 93 |
| Benhamou 2019 | **Intervention(s) and control(s)** |  |
|  | I | DBLG1 |
|  | C | the sensor-assisted pump therapy |
|  | Screened, n | 71 |
|  | **Randomized, n** |  |
|  | I | 33 |
|  | C | 35 |
|  | T | 68 |
|  | ITT, n | ITT population |
|  | Finishing study, n | 63 |
|  | Randomized finishing study (%) | -^d^ |
| Bergenstal 2019 | **Intervention(s) and control(s)** |  |
|  | I | either d-Nav and health-care professional support |
|  | C | health-care professional support |
|  | Screened, n | 236 |
|  | **Randomized, n** |  |
|  | I | 93 |
|  | C | 88 |
|  | T | 181 |
|  | ITT, n |  |
|  | **Finishing study, n** |  |
|  | I | 93 |
|  | C | 88 |
|  | T | 181 |
|  | **Randomized finishing study (%)** |  |
|  | I | 100 |
|  | C | 100 |
|  | T | 100 |
| Bretschneider 2022 | **Intervention(s) and control(s)** |  |
|  | I | using Vitadio |
|  | C | received standard diabetes care |
|  | Screened, n | 60 |
|  | Randomized, n | 60 (an intraindividual control group) |
|  | ITT, n |  |
|  | Finishing study, n | 42 |
|  | Randomized finishing study (%) | - |
| Charpentier 2011 | **Intervention(s) and control(s)** |  |
|  | I1 | electronic logbook |
|  | I2 | electronic logbook and telecousultation |
|  | C | usual paper logbook |
|  | Screened, n | 180 |
|  | **Randomized, n** |  |
|  | I1 | 60 |
|  | I2 | 59 |
|  | C | 61 |
|  | T | 180 |
|  | ITT, n |  |
|  | **Finishing study, n** |  |
|  | I1 | 56 |
|  | I2 | 57 |
|  | C | 60 |
|  | T | 163 |
|  | **Randomized finishing study (%)** |  |
|  | I1 | 93 |
|  | I2 | 97 |
|  | C | 98 |
|  | T | 91 |
| Franc 2019 | **Intervention(s) and control(s)** |  |
|  | I1 | interactive voice response system |
|  | I2 | Diabeo-BI app software |
|  | C | standard care |
|  | Screened, n | 189 |
|  | **Randomized, n** |  |
|  | I1 | 64 |
|  | I2 | 64 |
|  | C | 63 |
|  | T | 191 |
|  | ITT, n | ITT population |
|  | **Finishing study, n** |  |
|  | I1 | 63 |
|  | I2 | 64 |
|  | C | 62 |
|  | T | 189 |
|  | **Randomized finishing study (%)** |  |
|  | I1 | 98 |
|  | I2 | 98 |
|  | C | 100 |
|  | T | 99 |
| Franc 2020 | **Intervention(s) and control(s)** |  |
|  | I1 | DIABEO alone |
|  | I2 | DIABEO + telemonitoring by trained nurses |
|  | C | standard care |
|  | Screened, n | 665 |
|  | **Randomized, n** |  |
|  | I1 | 231 |
|  | I2 | 213 |
|  | C | 221 |
|  | T | 665 |
|  | ITT, n | ITT population |
|  | Finishing study, n | - |
|  | Randomized finishing study (%) | - |
| Guo 2021 | **Intervention(s) and control(s)** |  |
|  | I | received mHealth management |
|  | C | received their usual health management |
|  | Screened, n | 64 |
|  | **Randomized, n** |  |
|  | I | 32 |
|  | C | 32 |
|  | T | 64 |
|  | ITT, n | ITT population |
|  | **Finishing study, n** |  |
|  | I | 30 |
|  | C | 30 |
|  | T | 60 |
|  | **Randomized finishing study (%)** |  |
|  | I | 94 |
|  | C | 94 |
|  | T | 94 |
| Hsia 2022 | **Intervention(s) and control(s)** |  |
|  | I | BT-001 |
|  | C | a control app |
|  | Screened, n | 725 |
|  | **Randomized, n** |  |
|  | I | 326 |
|  | C | 343 |
|  | T | 669 |
|  | ITT, n | ITT population |
|  | **Finishing study, n** |  |
|  | I | 291 |
|  | C | 319 |
|  | T | 610 |
|  | **Randomized finishing study (%)** |  |
|  | I | 89 |
|  | C | 93 |
|  | T | 91 |
| Hsu 2016 | **Intervention(s) and control(s)** |  |
|  | I | received care through the cloud-based diabetes management program |
|  | C | received standard face-to-face care |
|  | Screened, n | 40 |
|  | **Randomized, n** |  |
|  | I | 20 |
|  | C | 20 |
|  | T | 40 |
|  | ITT, n |  |
|  | **Finishing study, n** |  |
|  | I | 19 |
|  | C | 16 |
|  | T | 35 |
|  | **Randomized finishing study (%)** |  |
|  | I | 95 |
|  | C | 80 |
|  | T | 88 |
| Jafar 2023 | **Intervention(s) and control(s)** |  |
|  | I | “Guru Diabetes” application |
|  | C | received standard care |
|  | Screened, n | 66 |
|  | **Randomized, n** |  |
|  | I | 33 |
|  | C | 33 |
|  | T | 66 |
|  | ITT, n |  |
|  | **Finishing study, n** |  |
|  | I | 29 |
|  | C | 33 |
|  | T | 62 |
|  | **Randomized finishing study (%)** |  |
|  | I | 88 |
|  | C | 100 |
|  | T | 94 |
| Lee 2018 | **Intervention(s) and control(s)** |  |
|  | I | tailored mobile coaching |
|  | C | regular information messages |
|  | Screened, n | 200 |
|  | **Randomized, n** |  |
|  | I | 74 |
|  | C | 74 |
|  | T | 148 |
|  | ITT, n |  |
|  | **Finishing study, n** |  |
|  | I | 72 |
|  | C | 64 |
|  | T | 136 |
|  | **Randomized finishing study (%)** |  |
|  | I | 97 |
|  | C | 86 |
|  | T | 92 |
| Lim 2022 | **Intervention(s) and control(s)** |  |
|  | I | the Nutritionist Buddy Diabetes (nBuddy Diabetes) mobile app |
|  | C | receiving standard diet counseling |
|  | Screened, n | 284 |
|  | **Randomized, n** |  |
|  | I | 72 |
|  | C | 76 |
|  | T | 148 |
|  | ITT, n |  |
|  | **Finishing study, n** |  |
|  | I | 67 |
|  | C | 73 |
|  | T | 140 |
|  | **Randomized finishing study (%)** |  |
|  | I | 93 |
|  | C | 96 |
|  | T | 95 |
| Moravcová 2022 | **Intervention(s) and control(s)** |  |
|  | I | used Vitadio |
|  | C | received a series of in-person consultations |
|  | Screened, n | 100 |
|  | **Randomized, n** |  |
|  | I | 50 |
|  | C | 50 |
|  | T | 100 |
|  | ITT, n |  |
|  | **Finishing study, 3-month/6-month, n** |  |
|  | I | 40/28 |
|  | C | 38/23 |
|  | T | 78/51 |
|  | **Randomized finishing study (%)** |  |
|  | I | 80/56 |
|  | C | 76/46 |
|  | T | 78/51 |
| Pamungkas 2022 | **Intervention(s) and control(s)** |  |
|  | I | received a 12-week smartphone application of diabetes coaching intervention |
|  | C | received the usual care from the community health centers |
|  | Screened, n | 60 |
|  | **Randomized, n** |  |
|  | I | 30 |
|  | C | 30 |
|  | T | 60 |
|  | ITT, n |  |
|  | **Finishing study,** **n** |  |
|  | I | 30 |
|  | C | 30 |
|  | T | 60 |
|  | **Randomized finishing study (%)** |  |
|  | I | 100 |
|  | C | 100 |
|  | T | 100 |
| Quinn 2008 | **Intervention(s) and control(s)** |  |
|  | I | BlueStar |
|  | C | received standard care |
|  | Screened, n | 30 |
|  | **Randomized, n** |  |
|  | I | 15 |
|  | C | 15 |
|  | T | 30 |
|  | ITT, n |  |
|  | **Finishing study,** **n** |  |
|  | I | 13 |
|  | C | 13 |
|  | T | 26 |
|  | **Randomized finishing study (%)** |  |
|  | I | 87 |
|  | C | 87 |
|  | T | 87 |
| Sachmechi 2023 | **Intervention(s) and control(s)** |  |
|  | I | using the Vivovitals diabetes platform |
|  | C | received usual clinical care |
|  | Screened, n | 130 |
|  | **Randomized, n** |  |
|  | I | 50 |
|  | C | 50 |
|  | T | 100 |
|  | ITT, n |  |
|  | **Finishing study,** **n** |  |
|  | I | 39 |
|  | C | 39 |
|  | T | 78 |
|  | **Randomized finishing study (%)** |  |
|  | I | 78 |
|  | C | 78 |
|  | T | 78 |
| Satish 2007 | **Intervention(s) and control(s)** |  |
|  | I | Personal digital assistant (PDA) |
|  | C | given a glucose meter and an unlimited supply of test strips for SMBG |
|  | Screened, n | 123 |
|  | **Randomized, n** |  |
|  | I | 61 |
|  | C | 60 |
|  | T | 121 |
|  | ITT, n |  |
|  | **Finishing study,** **n** |  |
|  | I | 61 |
|  | C | 60 |
|  | T | 121 |
|  | **Randomized finishing study (%)** |  |
|  | I | 100 |
|  | C | 100 |
|  | T | 100 |
| Stone 2010 | **Intervention(s) and control(s)** |  |
|  | I | ACM+HT group (active care management with home telemonitoring) |
|  | C | CC group (a monthly care coordination telephone call) |
|  | Screened, n | 1055 |
|  | **Randomized, n** |  |
|  | I | 73 |
|  | C | 77 |
|  | T | 150 |
|  | ITT, n |  |
|  | **Finishing study,** **n** |  |
|  | I | 64 |
|  | C | 73 |
|  | T | 137 |
|  | **Randomized finishing study (%)** |  |
|  | I | 88 |
|  | C | 95 |
|  | T | 91 |
| **Total** |  |  |
| All interventions | Randomized, n | 1729 |
|  | Finishing study, n | 1114 |
| All controls | Randomized, n | 1535 |
|  | Finishing study, n | 1145 |
| All interventions and controls | Randomized, n | 3264 |
|  | Finishing study, n | 2301 |

^a^Intervention group

^b^Control group

^c^Total

^d^Not available
