# Supplementary material for: Impact of Digital Therapeutics for the Management of Adult Patients With Diabetes: Systematic Review and Meta-Analysis of Randomized Controlled Trials
Source: J Med Internet Res. 2025 Sep 8;27:e70428. doi: 10.2196/70428 (PMC12455173; doi:10.2196/70428)
Supplement: Multimedia Appendix 5 [file jmir_v27i1e70428_app5.docx]

**Appendix 5. Baseline characteristics**

**Table1. Baseline characteristics (Ⅰ)**

| Study ID | Characteristic | Value |
| --- | --- | --- |
| Agarwal 2019 | **Intervention(s) and control(s)** |  |
|  | I^a^ | BlueStar (immediate treatment  group, ITG) |
|  | C^b^ | wait-list control group, WLC |
|  | **Gender, female, n (%)** |  |
|  | I | 48 (44.0) |
|  | C | 58 (51.0) |
|  | Total | 106 (48.0) |
|  | **Age, mean years (SD) or as stated** |  |
|  | I | 51.5 (10.6) |
|  | C | 52.1 (10.7) |
|  | Total | 51.8 (10.7) |
|  | **Ethnic groups, n (%)** |  |
|  | **I** |  |
|  | Caucasian | 46 (41.8) |
|  | Non-Caucasian | 64 (58.2) |
|  | **C** |  |
|  | Caucasian | 50 (44.3) |
|  | Non-Caucasian | 60 (53.0) |
| Benhamou 2019 | **Intervention(s) and control(s)** |  |
|  | I | DBLG1 |
|  | C | the sensor-assisted pump therapy |
|  | **Gender, female, n (%)** | 39 (62) |
|  | **Age, mean years (SD) or as stated** | 48.2 (13.4) |
|  | **Ethnic groups, n (%)** | -^c^ |
| Bergenstal 2019 | **Intervention(s) and control(s)** |  |
|  | I | either d-Nav and health-care professional support |
|  | C | health-care professional support |
|  | **Gender, female, n (%)** |  |
|  | I | 48 (52) |
|  | C | 45 (48) |
|  | **Age, mean years (SD) or as stated** |  |
|  | I | 61.7 (6.90) |
|  | C | 58.8 (8.5) |
|  | Total | 60.3 (7.9) |
|  | **Ethnic groups, n (%)** |  |
|  | **I** |  |
|  | American Indian or Alaska Native | 2 (2) |
|  | Asian | 2 (2) |
|  | African American | 19 (20) |
|  | Native Hawaiian or other Pacific Islander | 0 |
|  | White | 68 (73) |
|  | More than one | 0 |
|  | Unknown | 2 (2) |
|  | **C** |  |
|  | American Indian or Alaska Native | 2 (2) |
|  | Asian | 5 (6) |
|  | African American | 19 (22) |
|  | Native Hawaiian or other Pacific Islander | 0 |
|  | White | 57 (65) |
|  | More than one | 2 (2) |
|  | Unknown | 3 (3) |
| Bretschneider 2022 | **Intervention(s) and control(s)** |  |
|  | I | using Vitadio |
|  | C | received standard diabetes care |
|  | **Gender, female, n (%)** | 19 (45) |
|  | **Age, mean years (SD) or as stated** | 57 (7.4) |
|  | **Ethnic groups, n (%)** | - |
| Charpentier 2011 | **Intervention(s) and control(s)** |  |
|  | I1 | electronic logbook |
|  | I2 | electronic logbook and telecousultation |
|  | C | usual paper logbook |
|  | **Gender, female, n (%)** |  |
|  | I1 | 37 (71.7) |
|  | I2 | 37 (62.7) |
|  | C | 40 (65.6) |
|  | Total | 124 (63.3) |
|  | **Age, mean years (SD) or as stated** |  |
|  | I1 | 32.9 (11.7) |
|  | I2 | 31.6 (12.5) |
|  | C | 36.8 (14.1) |
|  | Total | 33.8 (12.9) |
|  | **Ethnic groups, n (%)** | - |
| Franc 2019 | **Intervention(s) and control(s)** |  |
|  | I1 | interactive voice response system |
|  | I2 | Diabeo-BI app software |
|  | C | standard care |
|  | **Gender, female, n (%)** |  |
|  | I1 | 41 (64.1) |
|  | I2 | 43 (68.3) |
|  | C | 38 (61.3) |
|  | Total | 122 (64.6) |
|  | **Age, mean years (SD) or as stated** |  |
|  | I1 | 58.1 (10.3) |
|  | I2 | 58.4 (9.2) |
|  | C | 59.6 (9.3) |
|  | Total | 58.7 (9.6) |
|  | **Ethnic groups** | 18 French hospitals |
| Franc 2020 | **Intervention(s) and control(s)** |  |
|  | I1 | DIABEO alone |
|  | I2 | DIABEO+ telemonitoring by trained nurses |
|  | C | standard care |
|  | **Gender, female, n(%)** |  |
|  | I1 | 157 (68) |
|  | I2 | 97 (45.5) |
|  | C | 111 (50.2) |
|  | Total | 342 (51.4) |
|  | **Age, mean years (SD) or as stated** |  |
|  | I1 | 39.1 (13.6) |
|  | I2 | 38.1 (13.4) |
|  | C | 38.3 (14.6) |
|  | Total | 38.5 (13.8) |
|  | **Ethnic groups** | different French regions |
| Guo 2021 | **Intervention(s) and control(s)** |  |
|  | I | received mHealth management |
|  | C | received their usual health management |
|  | **Gender, female, n(%)** |  |
|  | I | 9 (28.13) |
|  | C | 16 (50) |
|  | **Age, mean years (SD) or as stated** |  |
|  | I | 55.25 (13.8) |
|  | C | 59.46 (14.59) |
|  | **Ethnic groups** | from a general hospital in Hangzhou City, Zhejiang Province, China |
| Hsia 2022 | **Intervention(s) and control(s)** |  |
|  | I | BT-001 |
|  | C | a control app |
|  | **Gender, female, n (%)** |  |
|  | I | 176 (54) |
|  | C | 58 (8) |
|  | **Age, mean years (SD) or as stated** |  |
|  | I | 57 (9) |
|  | C | 58 (8) |
|  | **Ethnic groups, n (%)** |  |
|  | **I** |  |
|  | White | 189 (59) |
|  | Black or African American | 89 (28) |
|  | Asian | 13 (4) |
|  | American Indian or Alaskan Native | 3 (1) |
|  | Native Hawaiian or other Pacific Islander | 1 (0.3) |
|  | Other (includes multiple races) or not reported | 26 (8) |
|  | **C** |  |
|  | White | 201 (59) |
|  | Black or African American | 98 (29) |
|  | Asian | 15 (4) |
|  | American Indian or Alaskan Native | 3 (1) |
|  | Native Hawaiian or other Pacific Islander | 1 (0.3) |
|  | Other (includes multiple races) or not reported | 25 (7) |
| Hsu 2016 | **Intervention(s) and control(s)** |  |
|  | I | received care through the cloud-based diabetes |
|  | C | received standard face-to-face care |
|  | **Gender, female, n (%)** | - |
|  | **Age, mean years (SD) or as stated** |  |
|  | I | 53.3 |
|  | C | 53.8 |
|  | **Ethnic groups, n (%)** | - |
| Jafar 2023 | **Intervention(s) and control(s)** |  |
|  | I | "Guru Diabetes" application |
|  | C | received standard care |
|  | **Gender, female, n (%)** |  |
|  | I | 21 (72.4) |
|  | C | 27 (81.8) |
|  | **Age, mean years (SD) or as stated** |  |
|  | I | 54.24 (0.56) |
|  | C | 59.18 (10.24) |
|  | **Ethnic groups, n (%)** | - |
| Lee 2018 | **Intervention(s) and control(s)** |  |
|  | I | tailored mobile coaching |
|  | C | regular information messages |
|  | **Gender, female, n (%)** |  |
|  | I | 30 (41.7) |
|  | C | 20 (31.2) |
|  | **Age, mean years (SD) or as stated** |  |
|  | I | 51.4 (7.69) |
|  | C | 52.6 (7.9) |
|  | **Ethnic groups, n(%)** | - |
| Lim 2022 | **Intervention(s) and control(s)** |  |
|  | I | the Nutritionist Buddy Diabetes (nBuddy Diabetes) mobile app |
|  | C | receiving standard diet counseling |
|  | **Gender, female, n (%)** |  |
|  | I | 29 (40.3) |
|  | C | 30 (39.5) |
|  | Total | 59 (40) |
|  | **Age, mean years (SD) or as stated** |  |
|  | I | 51.9 (8.7) |
|  | C | 54.3 (9.9) |
|  | Total | 53.1 (9.3) |
|  | **Ethnic groups, n (%)** |  |
|  | **I** |  |
|  | Chinese | 57 (79.2) |
|  | Malay | 7 (9.7) |
|  | Indian | 7 (9.7) |
|  | Others | 1 (1.4) |
|  | **C** |  |
|  | Chinese | 50 (65.8) |
|  | Malay | 16 (21.1) |
|  | Indian | 5 (6.6) |
|  | Others | 5 (6.6) |
| Moravcová 2022 | **Intervention(s) and control(s)** |  |
|  | I | used Vitadio |
|  | C | received a series of in-person consultations |
|  | **Gender, female, n (%)** | - |
|  | **Age, mean years (SD) or as stated** | - |
|  | **Ethnic groups** | speak fluently Czech |
| Pamungkas 2022 | **Intervention(s) and control(s)** |  |
|  | I | received a 12-week smartphone application of diabetes coaching intervention |
|  | C | received the usual care from the community health centers |
|  | **Gender, female, n (%)** |  |
|  | I | 24 (80) |
|  | C | 19 (63.3) |
|  | **Age, mean years (SD) or as stated** |  |
|  | I | 56.2 (7.63) |
|  | C | 54.5 (9.20) |
|  | **Ethnic groups, n (%)** | - |
| Quinn 2008 | **Intervention(s) and control(s)** |  |
|  | I | BlueStar |
|  | C | received standard care |
|  | **Gender, female, n (%)** |  |
|  | I | 9 (69.23) |
|  | C | 8 (61.54) |
|  | **Age, mean years (SD) or as stated** |  |
|  | Total | 51.05 (11.03) |
|  | **Ethnic groups, n (%)** |  |
|  | **I** |  |
|  | African American | 10 (76.92) |
|  | White (non-Hispanic) | 3 (23.08) |
|  | **C** |  |
|  | African American | 6 (46.15) |
|  | White (non-Hispanic) | 7 (53.85) |
| Sachmechi 2023 | **Intervention(s) and control(s)** |  |
|  | I | using the Vivovitals diabetes platform |
|  | C | received usual clinical care |
|  | **Gender, female, n (%)** |  |
|  | I | (44.7) |
|  | C | (56.6) |
|  | **Age, mean years (SD) or as stated** |  |
|  | I | 58.9 (10.3) |
|  | C | 64.5 (13.6) |
|  | **Ethnic groups, n (%)** |  |
|  | **I** |  |
|  | Asian | 24 (61.54) |
|  | White | 2 (5.13) |
|  | Black/AA | 11 (28.21) |
|  | Hispanic | 1 (2.56) |
|  | Not specified | 1 (2.56) |
|  | **C** |  |
|  | Asian | 19 (48.72) |
|  | White | 5 (12.82) |
|  | Black/AA | 11 (28.21) |
|  | Hispanic | 3 (7.69) |
|  | Not specified | 1 (2.56) |
| Satish 2007 | **Intervention(s) and control(s)** |  |
|  | I | Personal digital assistant (PDA) |
|  | C | given a glucose meter and an unlimited supply of test strips for SMBG |
|  | **Gender, female, n (%)** |  |
|  | I | 31 (51.67) |
|  | C | 34 (55.74) |
|  | **Age, mean years (SD) or as stated** |  |
|  | I | 33.0 |
|  | C | 32.5 |
|  | **Ethnic groups, n (%)** |  |
|  | **I** |  |
|  | Caucasian | 56 (91.8) |
|  | Other | 5 (8.2) |
|  | **C** |  |
|  | Caucasian | 56 (93.3) |
|  | Other | 4 (6.7) |
| Stone 2010 | **Intervention(s) and control(s)** |  |
|  | I | ACM+HT group (active care management with home telemonitoring) |
|  | C | CC group (a monthly care coordination telephone call) |
|  | **Gender, female, n (%)** | The vast majority were male |
|  | **Age, mean years (SD) or as stated** | One-third of the participants in both groups ≥ 65 |
|  | **Ethnic groups** | the vast majority were non-Hispanic white |

^a^Intervention group

^b^Control group

^c^Not available

**Table2. Baseline characteristics(Ⅱ)**

| Study ID | Characteristic | Value |
| --- | --- | --- |
| Agarwal 2019 | **Intervention(s) and control(s)** |  |
|  | I^a^ | BlueStar (immediate treatment  group, ITG) |
|  | C^b^ | wait-list control group, WLC |
|  | **Duration of disease, mean years (SD) or as stated, n** |  |
|  | **I** |  |
|  | 0-6 months | 16 |
|  | > 6 months to 2 years | 25 |
|  | > 2-5 years | 26 |
|  | > 5 years | 41 |
|  | Unsure | 1 |
|  | Missing | 1 |
|  | **C** |  |
|  | 0-6 months | 24 |
|  | > 6 months to 2 years | 27 |
|  | > 2-5 years | 13 |
|  | > 5 years | 47 |
|  | Unsure | 2 |
|  | Missing | 0 |
|  | **HbA1c (%), mean (SD)** |  |
|  | I | 8.89 (1.82) |
|  | C | 9.03 (1.53) |
|  | Total | 8.96 (1.68) |
|  | BMI (kg/ m^2^), mean (SD) | -^c^ |
|  | Duration of intervention | 6 months |
| Benhamou 2019 | **Intervention(s) and control(s)** |  |
|  | I | DBLG1 |
|  | C | the sensor-assisted pump therapy |
|  | **Duration of disease, mean years (SD) or as stated** | 28.0 (13.6) years |
|  | **HbA1c (%), mean (SD)** | 7.6 (0.9) |
|  | BMI (kg/ m^2^), mean (SD) | 24.8 (3.5) |
|  | Duration of intervention | 12 weeks |
| Bergenstal 2019 | **Intervention(s) and control(s)** |  |
|  | I | either d-Nav and health-care professional support |
|  | C | health-care professional support |
|  | **Duration of disease, mean years (SD) or as stated** |  |
|  | I | 16.1 (7.3) years |
|  | C | 15.3 (6.0) years |
|  | **HbA1c (%), mean (SD)** |  |
|  | I | 8.7 (0.8) |
|  | C | 8.5 (0.8) |
|  | **BMI (kg/ m^2^), mean (SD)** |  |
|  | I | 34.7 (5.1) |
|  | C | 34.9 (5.0) |
|  | Duration of intervention | 6 months |
| Charpentier 2011 | **Intervention(s) and control(s)** |  |
|  | I1 | electronic logbook |
|  | I2 | electronic logbook and telecousultation |
|  | C | usual paper logbook |
|  | **Duration of disease, mean years (SD) or as stated** | > 1 year |
|  | **HbA1c (%), mean (SD)** |  |
|  | I1 | 9.19 (1.14) |
|  | I2 | 9.11 (1.14) |
|  | C | 8.91 (0.90) |
|  | Total | 9.07 (1.07) |
|  | **BMI (kg/ m^2^), mean (SD)** |  |
|  | I1 | 23.8 (3.3) |
|  | I2 | 25.8 (5.0) |
|  | C | 25.1 (6.8) |
|  | Total | 24.9 (5.2) |
|  | Duration of intervention | 6 months |
| Franc 2019 | **Intervention(s) and control(s)** |  |
|  | I1 | interactive voice response system |
|  | I2 | Diabeo-BI app software |
|  | C | standard care |
|  | **Duration of disease, mean years (SD) or as stated** | > 3 years |
|  | **HbA1c (%), mean (SD)** |  |
|  | I1 | 8.9 (1.1) |
|  | I2 | 9.0 (1.0) |
|  | C | 8.9 (1.2) |
|  | Total | 8.9 (1.1) |
|  | **BMI (kg/ m^2^), mean (SD)** |  |
|  | I1 | 28.9 (4.7) |
|  | I2 | 30.4 (4.6) |
|  | C | 29.7 (5.9) |
|  | Total | 29.7 (5.1) |
|  | Duration of intervention | 4 months |
| Franc 2020 | **Intervention(s) and control(s)** |  |
|  | I1 | DIABEO alone |
|  | I2 | DIABEO + telemonitoring by trained nurses |
|  | C | standard care |
|  | **Duration of disease, mean years (SD) or as stated** | > 15 months, the mean diabetes duration following diagnosis was 17.4 years |
|  | **HbA1c (%), mean (SD)** |  |
|  | I1 | 9.1 (1.1) |
|  | I2 | 9.1 (0.9) |
|  | C | 9.1 (1.0) |
|  | Total | 9.1 (1.0) |
|  | **BMI (kg/ m^2^), mean (SD)** |  |
|  | I1 | 25.8 (4.7) |
|  | I2 | 26.1 (4.7) |
|  | C | 26.0 (5.0) |
|  | Total | 26.0 (4.8) |
|  | Duration of intervention | 12 months |
| Guo 2021 | **Intervention(s) and control(s)** |  |
|  | I | received mHealth management |
|  | C | received their usual health management |
|  | **Duration of disease, mean years (SD) or as stated** | - |
|  | **HbA1c (%), mean (SD)** |  |
|  | I | 8.97 (2.12) |
|  | C | 9.14 (1.96) |
|  | **BMI (kg/ m^2^), mean (SD)** |  |
|  | I | 24.00 (3.13) |
|  | C | 24.32 (3.14) |
|  | Duration of intervention | 4 weeks |
| Hsia 2022 | **Intervention(s) and control(s)** |  |
|  | I | BT-001 |
|  | C | a control app |
|  | **Duration of disease, mean years (SD) or as stated** |  |
|  | I | 11 (8) years |
|  | C | 11 (8) years |
|  | **HbA1c (%), mean (SD)** |  |
|  | I | 8.2 (0.1) |
|  | C | 8.1 (0.1) |
|  | **BMI (kg/ m^2^), mean (SD)** |  |
|  | I | 35 (7) |
|  | C | 35 (7) |
|  | Duration of intervention | 3 months |
| Hsu 2016 | **Intervention(s) and control(s)** |  |
|  | I | received care through the cloud-based diabetes management program |
|  | C | received standard face-to-face care |
|  | **Duration of disease, mean years (SD) or as stated** |  |
|  | I | 9.6 years |
|  | C | 9.0 years |
|  | **HbA1c (%), mean (SD)** |  |
|  | I | 10.8 |
|  | C | 10.9 |
|  | **BMI (kg/ m^2^), mean (SD)** |  |
|  | I | 30.8 |
|  | C | 31.7 |
|  | Duration of intervention | 12 ±2 weeks |
| Jafar 2023 | **Intervention(s) and control(s)** |  |
|  | I | “Guru Diabetes” |
|  | C | received standard care |
|  | **Duration of disease, mean years (SD) or as stated** |  |
|  | I | 6.93 (4.86) years |
|  | C | 7.42 (4.78) years |
|  | **HbA1c (%), mean (SD)** |  |
|  | I | 9.56 (1.95) |
|  | C | 8.66 (1.69) |
|  | BMI (kg/ m^2^), mean (SD) | - |
|  | Duration of intervention | 3 months |
| Lee 2018 | **Intervention(s) and control(s)** |  |
|  | I | tailored mobile coaching |
|  | C | regular information messages |
|  | **Duration of disease, mean years (SD) or as stated** |  |
|  | I | 7.5 (4.8) years |
|  | C | 9.3 (6.0) years |
|  | **HbA1c (%), mean (SD)** |  |
|  | I | 8.1 (1.5) |
|  | C | 8.0 (1.2) |
|  | **BMI (kg/ m^2^), mean (SD)** |  |
|  | I | 26.1 (3.3) |
|  | C | 26.3 (3.2) |
|  | Duration of intervention | 6 months |
| Lim 2022 | **Intervention(s) and control(s)** |  |
|  | I | the Nutritionist Buddy Diabetes (nBuddy Diabetes) mobile app |
|  | C | receiving standard diet counseling |
|  | **Duration of disease, mean years (SD) or as stated** |  |
|  | I | 1.9 (2.3) years |
|  | C | 2.4 (2.6) years |
|  | Total | 2.2 (2.5) years |
|  | **HbA1c (%), mean (SD)** |  |
|  | I | 5.94 (0.48) |
|  | C | 6.06 (0.50) |
|  | **BMI (kg/ m^2^), mean (SD)** |  |
|  | I | 29.8 (4.2) |
|  | C | 29.8 (3.9) |
|  | Total | 29.8 (4.1) |
|  | Duration of intervention | 6 months |
| Moravcová 2022 | **Intervention(s) and control(s)** |  |
|  | I | used Vitadio |
|  | C | received a series of in-person consultations |
|  | Duration of disease, mean years (SD) or as stated | diagnosed type 2 diabetes mellitus or prediabetes (fasting glucose within the range of 5.6–6.9 mmol/L or oral glucose tolerance test (OGTT) within the range of 7.8–11.0 mmol/L) or insulin resistance (IR) (defined as HOMA-IR > 2.7) |
|  | **HbA1c (%), mean (SD)** |  |
|  | I | 7.20 (1.30) |
|  | C | 6.70 (1.90) |
|  | BMI (kg/ m^2^), mean (SD) | - |
|  | Duration of intervention | 3 months |
| Pamungkas 2022 | **Intervention(s) and control(s)** |  |
|  | I | received a 12-week smartphone application of diabetes coaching intervention |
|  | C | received the usual care from the community health centers |
|  | Duration of disease, mean years (SD) or as stated | > 2 years |
|  | **HbA1c (%), mean (SD)** |  |
|  | I | 8.04 (1.96) |
|  | C | 8.55 (2.95) |
|  | **BMI (kg/ m^2^), mean (SD)** |  |
|  | I | 23.70 (3.53) |
|  | C | 24.32 (3.51) |
|  | Duration of intervention | 12 weeks |
| Quinn 2008 | **Intervention(s) and control(s)** |  |
|  | I | BlueStar |
|  | C | received standard care |
|  | **Duration of disease, mean years (SD) or as stated** |  |
|  | I | 7.61 years |
|  | C | 11 years |
|  | **HbA1c (%), mean (SD)** |  |
|  | I | 9.51 |
|  | C | 9.05 |
|  | **BMI (kg/ m^2^), mean (SD)** |  |
|  | I | 34.07 |
|  | C | 34.58 |
|  | Duration of intervention | 3 months |
| Sachmechi 2023 | **Intervention(s) and control(s)** |  |
|  | I | using the Vivovitals diabetes platform |
|  | C | received usual clinical care |
|  | **Duration of disease, mean years (SD) or as stated** |  |
|  | I | 13.9 (7.5) years |
|  | C | 15.4 (8.0) years |
|  | **HbA1c (%), mean (SD)** |  |
|  | I | 9.5 (1.7) |
|  | C | 9.6 (1.4) |
|  | **BMI (kg/ m^2^), mean (SD)** |  |
|  | I | 33.6 |
|  | C | 30.9 |
|  | Duration of intervention | 12 weeks |
| Satish 2007 | **Intervention(s) and control(s)** |  |
|  | I | Personal digital assistant (PDA) |
|  | C | given a glucose meter and an unlimited supply of test strips for SMBG |
|  | **Duration of disease, mean years (SD) or as stated** |  |
|  | I | 17.1 years |
|  | C | 17.3 years |
|  | HbA1c (%), mean (SD) | - |
|  | **BMI (kg/ m^2^), mean (SD)** |  |
|  | I | 26.2 |
|  | C | 27.2 |
|  | Duration of intervention | 12 months |
| Stone 2010 | **Intervention(s) and control(s)** |  |
|  | I | ACM+HT group (active care management with home telemonitoring) |
|  | C | CC group (a monthly care coordination telephone call) |
|  | Duration of disease, mean years (SD) or as stated | ≥ 12 months |
|  | **HbA1c (%), mean (SD)** |  |
|  | I | 9.60 (1.60) |
|  | C | 9.40 (1.40) |
|  | BMI (kg/ m^2^), mean (SD) | - |
|  | Duration of intervention | 6 months |

^a^Intervention group

^b^Control group

^c^Not available
