# Supplementary material for: Impact of Digital Therapeutics for the Management of Adult Patients With Diabetes: Systematic Review and Meta-Analysis of Randomized Controlled Trials
Source: J Med Internet Res. 2025 Sep 8;27:e70428. doi: 10.2196/70428 (PMC12455173; doi:10.2196/70428)
Supplement: Multimedia Appendix 6 [file jmir_v27i1e70428_app6.docx]

**Appendix 6. Description of interventions**

| **Characteristic**  **Study ID** | **Characteristic**  **Study ID** | **Registered** | **Intervention(s)** | **Control(s)** |
| --- | --- | --- | --- | --- |
| **Stone 2010** | **Agarwal 2019** | Yes | The intervention was the BlueStar mobile app, designed to act as a virtual coach for patients with T2DM. The app was preloaded onto a cellular network–connected Samsung smartphone (with all other features disabled). The phone was connected to a cellular data plan for internet connectivity and was able to connect to local Wi-Fi networks. If participants used the app without an internet connection, the information was saved and uploaded to the secure server when the phone regained an internet connection. Patients could enter information related to T2DM management into the app, including baseline health, daily blood glucose readings, exercise activity, and food intake. The app used this information to deliver customized, evidence-based messages in real time that aim to impact motivation, behavior, and education. The messages, based on the Transtheoretical Model of Behavior Change, included educational and affirmational content to encourage sustained behavior changes. Educational messages were aligned with the American Association of Diabetes Educators 7 Standard of Care. The app also facilitated the transfer of data to the user’s clinician through Smart Visit reports that provide a clinical overview of current diabetes management including recent blood sugar readings. | Patients in the WLC group received usual diabetes care by the DEP and their primary care physician for the first 3 months of the study. To align with the principles of pragmatic trials, the usual care received was not standardized among participants. |

**Appendix4 (Continued)**

| **Characteristic**  **Study ID** | **Characteristic**  **Study ID** | **Registered** | **Intervention(s)** | **Control(s)** |
| --- | --- | --- | --- | --- |
| **Stone 2010** | **Benhamou 2019** | Yes | Patients assigned to the DBLG1 group in the first 12 week treatment period used the Cellnovo insulin patch-pump managed by the Diabeloop application installed on an android smartphone and connected to the Dexcom G5 continuous glucose monitoring system using Bluetooth Low Energy technology. At the start of the closed-loop period, at the request of the ANSM, patients were admitted to the local hospital research facility for 48 h to receive training on closed-loop insulin delivery, whereby a dedicated nurse taught patients how to use the various components of the system (sensor, pump, smartphone) and how to respond to an alarm. The nurse was then responsible for remote monitoring and phone interaction with the given patient. Remote monitoring was implemented at the request of the ANSM. Customisation of the closed-loop system required it to be tuned through eight settings, which was done during this initial 48 h stay. The DBLG1 system, which combines an algorithm based on machine-learning within a physiological framework with an expert system and self-learning algorithms, is a hybrid closed-loop device that requires the patient to record carbohydrate intake semi-quantitatively, and intensity and duration of planned physical activities.  In both the DBLG1 and sensor-assisted pump therapy groups, hospital visits were scheduled at weeks 1, 3, 6, 9, and 12 to download data from the command terminal or Dexcom receiver, to monitor adverse events, and to complete satisfaction questionnaires. | Patients assigned to the sensor-assisted pump therapy group returned to their usual treatment with their own pump, combined with a Dexcom G5 Mobile continuous glucose monitoring system (Dexcom, San Diego, CA, USA). Participants were free to activate or shut off sensor alarms and no recommended thresholds were used for high-glucose and low-glucose alarms.  In both the DBLG1 and sensor-assisted pump therapy groups, hospital visits were scheduled at weeks 1, 3, 6, 9, and 12 to download data from the command terminal or Dexcom receiver, to monitor adverse events, and to complete satisfaction questionnaires. |

**Appendix4 (Continued)**

| **Characteristic**  **Study ID** | **Characteristic**  **Study ID** | **Registered** | **Intervention(s)** | **Control(s)** |
| --- | --- | --- | --- | --- |
| **Stone 2010** | **Bergenstal 2019** | Yes | Participants who were randomly assigned to the intervention group were provided with and trained on the use of the d-Nav device. The d-Nav device was then set up with the participant’s current insulin regimen and dosage. Participants in both groups received free testing supplies either for d-Nav (intervention group) or for their glucose meters’ consumables (control group).  Participants assigned to the intervention group were also asked about details pertaining to the use of the device by use of a preprepared follow-up form, which was filled in by the health-care professional. | Participants were followed up for 6 months, during which participants in both groups had seven health-care professional–patient interactions (three face-to-face and four phone visits) with the study team who inquired about participants’ wellbeing, health changes, challenges in management, and any side-effects; no specific questionnaire was followed. |
|  | **Bretschneider 2022** | No | Vitadio is a digital care program designed to empower patients with effective self-management and lifestyle change. It consists of a three-month intensive phase followed by a sustained phase. The mobile application guides patients throughout the program using a system of daily tasks and automated messages. Patients follow educational courses, including topics ranging from motivation to diet, physical activity, sleep hygiene, mental wellbeing, and social aspects of life with diabetes. Personal weekly goals help to select relevant habits and track them daily. The Vitadio app enables monitoring of metabolic (e.g., body weight, waist circumference, glycemia) and lifestyle (e.g., steps, diet, mood) parameters. To track dietary habits, the patients can use a feature designated to upload photos of their meals. The program is enhanced by a set of communication features employing human support. To ensure patient safety and enhance effective use of the program, a personal advisor is available by chat to answer patient questions. To improve adherence, patients can participate in a peer support group. Vitadio complements therapy set by a physician and is certified as a class I medical device. | The Control group consists of retrospective observations of the same participants from the intervention group. Therefore, HbA1c values measured 3 months before the start of the study were collected retrospectively. The control group received standard diabetes care as defined by the German diabetes disease management program and provided by diabetes specialists and/or general practitioner in Germany. |

**Appendix4 (Continued)**

| **Characteristic**  **Study ID** | **Characteristic**  **Study ID** | **Registered** | **Intervention(s)** | **Control(s)** |
| --- | --- | --- | --- | --- |
| **Stone 2010** | **Charpentier 2011** | Yes | Participants randomized to group G2 received a smartphone loaded with the Diabeo software. They did not use the teleconsultation option, but face-to-face follow-up visits were planned for month 3 and month 6.  Participants randomized to group G3 received a smartphone with the Diabeo software. No follow-up hospital visits were scheduled, until end point at month 6, but teleconsultations by telephone call were planned every 2 weeks. Participant SMPG, diet, and insulin treatment data were automatically uploaded by the smartphone to a secured website, where they were available to investigators at any time, including during the teleconsultations. Teleconsultations were conducted with both patients and doctors in front of their computers or smartphone displaying last weeks’ data and focused on insulin dose adjustments and motivational support. Randomization was carried out using a Web-based system.  Diabeo software is a bolus calculator with validated algorithms, taking into account SMPG level before meals, carbohydrate counts, and planned physical activity. Parameters personally tailored for adjustment of prandial and basal insulin dose are entered into the system for each patient. If fasting or postprandial SMPG do not meet target levels, the system can suggest adjustments for carbohydrate ratio, long-acting insulin analog dose, or pump basal rates. | Participants in the control group (G1) had no electronic logbook but kept their paper logbook and were asked to attend two follow-up visits at the hospital, after 3 and 6 months. |
|  | **Franc 2019** | No | G2: IVRS + short telephone consultations + Face-to-face visit at M4  G3: DiabeoBI + short telephone consultations + Face-to-face visit at M4 | G1: Standard care + Face-to- face visit at M4 and optional visit at M1 |

**Appendix4 (Continued)**

| **Characteristic**  **Study ID** | **Characteristic**  **Study ID** | **Registered** | **Intervention(s)** | **Control(s)** |
| --- | --- | --- | --- | --- |
| **Stone 2010** | **Franc 2020** | Yes | Arm 2 (DIABEO® alone) and Arm 3 (DIABEO® + telemonitoring by trained nurses).  The protocol for delegating telemonitoring to the nursing team (arm 3) starts with the investigator physician who sets: (i) glycemic targets and associated treatment, (ii) alarm values that trigger nursing actions, and (iii) values for patient's self-adaptations. Then, a reference nurse initiates the patient to the use of the DIABEO app on his smartphone. The patient enters relevant data (glycemia, physical activity, and ingested carbohydrates) and DIABEO calculates the insulin dose (an eventual dose adaptations). These data are sent every 2 h to a platform that is continuously visible by the reference nurse and the investigator. Automatic messages containing analytical data are produced every night. These messages are analyzed by the reference nurse during the morning of each working day. Finally, the investigator receives the data from the patients and the reports from the nurses.  Following a screening period of 10 days, the main study period lasted 12 months, with an optional extension period of at least 12 additional months. | arm 1 (standard care)  If desired, patients from the control group could start using DIABEO® after 12 months. |

**Appendix4 (Continued)**

| **Characteristic**  **Study ID** | **Characteristic**  **Study ID** | **Registered** | **Intervention(s)** | **Control(s)** |
| --- | --- | --- | --- | --- |
| **Stone 2010** | **Guo 2021** | No | Patients in the intervention group received mHealth management based on the mHealth management model that consisted of the network platform, an implantable glucose sensor, a mobile app and GP support. First, the implantable glucose sensor was subcutaneously implanted to enable patients to monitor their blood glucose at any time. Second, both patients and GPs downloaded the mobile app. Third, the patients filled in the health information retrospectively at any time of the day. Forth, the processor combined the dynamic trend graph with the information on lifestyle changes to analyse the causes of any blood glucose fluctuations. Finally, GPs received real-time information from patients and the processor in order to formulate personalised intervention programmes for their patients. | Patients in the control group received their usual health management. First, GPs conducted telephone followups once a week to obtain patients’ blood glucose values and urged patients to go to the outpatient clinic for their review on time. Then, public health education material for T2DM were given out at the followup visits to increase relevant knowledge about T2DM. The materials comprised four themes, including basic knowledge of T2DM, reasonable diets for T2DM, exercise therapy for T2DM and the prevention of T2DM complications. |

**Appendix4 (Continued)**

| **Characteristic**  **Study ID** | **Characteristic**  **Study ID** | **Registered** | **Intervention(s)** | **Control(s)** |
| --- | --- | --- | --- | --- |
| **Stone 2010** | **Hsia 2022** | Yes | Each week, BT-001 would ask subjects to complete a new behavioral module along with one or more related skillbased exercises. Modules addressed topics such as: personal beliefs and barriers (e.g., those related to a subject’s ability to change and control his or her behaviors); beliefs about macronutrients and the importance of various food types; hedonicrelated beliefs about pleasant or unpleasant sensations experienced by eating or exercising; and beliefs about exercise.  BT-001 is intended to be prescribed for use between clinic visits within 90-day treatment cycles, each of which includes 13 modules. The CBT content for the second treatment cycle was available during the first treatment cycle if subjects chose to work ahead. They were not required to complete any specific number of modules. BT-001–allocated subjects were asked to record information about their diet and exercise behaviors, perceptions, and beliefs, self-measured blood pressure, and glucose levels within the app. Based on these factors, the app presented a treatment plan summarizing daily and weekly goals to improve their glycemic control. | A control app |

**Appendix4 (Continued)**

| **Characteristic**  **Study ID** | **Characteristic**  **Study ID** | **Registered** | **Intervention(s)** | **Control(s)** |
| --- | --- | --- | --- | --- |
| **Stone 2010** | **Hsu 2016** | No | Subjects met with their HCPs during the initial visit. As in the control group, the HCP team comprised an endocrinologist and certified diabetes educators.  Each subject received a tablet computer at the initial visit. The diabetes management program was preloaded on the subject’s tablet computer with the medication regimen and the initial insulin dose. During the same visit, instructions on using the features and communication tools on the tablet computer were given. A glucose meter that was wirelessly connected to the tablet computer was also given to each subject. Subjects were instructed to perform self-monitoring of blood glucose once a day in the morning with the general goal of achieving a fasting glucose level between 80 and 110 mg/dL. As with the control group, subjects received education on insulin injection according to standard protocol at the center. They were also familiarized with the PREDICTIVE 303 protocol and hypoglycemia treatment guideline. No face-to-face appointments were scheduled during the study period until the end of the study. Subjects had virtual interactions with the HCPs on a regular basis as needed. | Subjects in the control group received standard care at the clinic in initiating and titrating insulin, with interim faceto-face visits, as well as telephone/fax communication with educators and physicians as dictated by their HCPs. The starting dosage and insulin titration schedule were determined by their HCPs. As part of routine care, the subjects were instructed to contact their HCPs if they experienced any hypoglycemic episode. For the duration of this study, HCPs were requested not to make changes in non–insulin diabetes medications. At the conclusion of 12 weeks, control subjects returned to the center to repeat the HbA1c test, anthropometric measurements, and DTSQ. Rates of hypoglycemia and the frequency of communications were obtained by reviewing the subjects’ medical records. |

**Appendix4 (Continued)**

| **Characteristic**  **Study ID** | **Characteristic**  **Study ID** | **Registered** | **Intervention(s)** | **Control(s)** |
| --- | --- | --- | --- | --- |
| **Stone 2010** | **Jafar 2023** | No | All participants were instructed to download the application of “Guru Diabetes” application. The coach monitored the data on participants’ dietary habits, physical activity, drug adherence, and routine measurements of blood pressure and blood glucose during the coaching period. All participants were provided with basic diabetes education through short videos available on the application. Participants were instructed to record their dietary habits, physical activity, drug adherence, and routine measurements of blood pressure and blood glucose in the application on a daily basis. Patients were instructed to send photos of their diet through WhatsApp, and were able to send messages to the coach 24 h a day. Small group video calls were conducted every 2weeks and in-person voice call was arranged as needed based on the data input in the application. | The control group received standard care from their clinic. |
|  | **Lee 2018** | No | In phase 1 of the study, participants in the I-M group received TMC, while participants in the C-I group maintained their usual diabetes care. After 6 months, phase 2 of the study was conducted and included the subjects who agreed to participate. During the second 6-month phase 2, participants in the C-I group received TMC for diabetes management. Participants in the I-M group could also access the Switch application, but received only regular messages regarding seasonal and health information without individualized message feedback by healthcare professionals..Users could upload their lifestyle and medical information, such as self-monitoring of blood glucose (SMBG), BP, exercise, dietary record, medication record, and body weight, and set their lifestyle goals automatically presented using algorithms based on self-reported behavioral habits. At any time, users could check their data by logging into the Switch application where they could obtain information on diabetes and other metabolic diseases. The data entered in the Switch application were automatically transmitted by wireless network to the server and stored on a secure website accessible only to providers. | |

**Appendix4 (Continued)**

| **Characteristic**  **Study ID** | **Characteristic**  **Study ID** | **Registered** | **Intervention(s)** | **Control(s)** |
| --- | --- | --- | --- | --- |
| **Stone 2010** | **Lim 2022** | No | Additionally, participants in the intervention group were introduced to the Nutritionist Buddy Diabetes (nBuddy Diabetes) mobile app during the baseline visit. They were required to download the nBuddy Diabetes app and educated to self-monitor their weight, diet, physical activity, and blood glucose levels for 6 months. The nBuddy Diabetes app is designed with an in-built algorithm that incorporates behavioral strategies to empower individuals through prompts and cues. These behavioral strategies include goal-setting, stimulus control, problem solving, self-monitoring, cognitive restructuring and motivational interviewing. The app’s automated response system evaluates the suitability of food choices and provides instantaneous feedback to generate a list of healthier and culturally appropriate food alternatives. The app provided an automated individualized calorie limit which was computed based on body weight, gender, age and activity level. The total daily carbohydrate intake was restricted to 40% of total daily calories. Participants were encouraged to log their meals via the app, with the goal of keeping within the preset calorie and carbohydrate limits. As part of the in-app features, self-monitoring of step count using the phone pedometer and physical activity steps conversion function, allowed participants to track their daily step counts. The app automatically set a gradual increase in step count goal starting from 3,000 in the first week to 10,000 steps per day by the third week of the program. Self-monitoring of weight loss progression and blood glucose level is enabled via the weight and blood glucose logging functions. Participants in the intervention group were advised to monitor and log their weight in the app twice weekly. A glucometer (FreeStyle Optium Neo, United Kingdom) for weekly blood glucose monitoring was also provided. In-app educational videos on weight management, diabetes prevention, healthy meal planning, carbohydrate foods in relation to glycemic response, behavioral strategies, and physical activity were uploaded for each participant on a weekly basis in the first 12 weeks. | The control group received standard face-to-face dietary advice based on healthy food plate meal-planning principles by a research dietitian. They were provided with a digital weighing scale for self-monitoring of their body weight. All participants were also encouraged to engage in 150 min per week of moderate intensity physical activity. |

**Appendix4 (Continued)**

| **Characteristic**  **Study ID** | **Characteristic**  **Study ID** | **Registered** | **Intervention(s)** | **Control(s)** |
| --- | --- | --- | --- | --- |
| **Stone 2010** | **Moravcová 2022** | Yes | The intervention group received the Vitadio app without any in-person lifestyle consultation. Vitadio is a certified class I medical device employing a multimodal therapy approach to provide individualized support in lifestyle modification and self-management. The digital care program consists of a 3-month intensive phase followed by a 3-month sustaining phase. Participants were able use the app for 12 months to maintain access to their logged data. The application guides patients through the program using a system of daily tasks and automated messages. The tasks develop according to the patient’s choices and progress in the program. Tasks focus on establishing a healthy routine, and their completion is positively reinforced by gamification principles. Patients follow an interactive educational course covering topics including motivation, healthy eating patterns, physical activity, sleep hygiene, mental wellbeing and social aspects of life with diabetes. The lessons are implemented using gamified personal goals that help patients root important habits into their daily life. Patients are also nudged to monitor their physiological and lifestyle parameters. The program is enhanced by a set of human support features. To ensure patient safety and enhance effective use of the program, a qualified personal dietitian was available on chat to answer patient questions. Optionally, participants were able to book an onboarding phone consultation with their dietitian. To improve adherence, patients can join a peer support group to share encouragement and experience. | The control group received 5 in-person lifestyle consultations over the course of 6 months. In the subsequent 6 months, participants were able to reach out to their educator for additional support in sustaining their lifestyle. The education was provided by a physician, dietitian and/or educational nurse at the Department of Exercise Medicine and Cardiovascular Rehabilitation. Additionally, participants received an online diary tool for recording meals, which included macronutrients composition analysis, energy intake calculation, recipes and sample diet plans. |

**Appendix4 (Continued)**

| **Characteristic**  **Study ID** | **Characteristic**  **Study ID** | **Registered** | **Intervention(s)** | **Control(s)** |
| --- | --- | --- | --- | --- |
| **Stone 2010** | **Pamungkas 2022** | No | The intervention group received the smartphone application of diabetes coaching intervention. The program consisted of 5 main menus: narrative App-based coaching, a printed user guide, mindfulnessbased coaching, skill-based coaching, and a small App-interaction.The smartphone application of diabetes coaching intervention is a personalized coaching program delivered via a mobile phone. Several sub-menus contained knowledge and information to assist T2DM on healthy lifestyle in daily living. Contents of the sub-menus emphasized DSM practice, such as lists of healthy foods and a daily portion of food intake, types of physical activity to fit with T2DM patients, medication adherence, and blood glucose monitoring. Moreover, foot care and peripheral neuropathy screening are also included in the mobile application.Participants were encouraged to record their self-management activities by filling the reporting menu of the application. Online consultation via Zoom meeting and a telephone call or line call was conducted to track the progress of DSM practice, monitor the possible complications, and solve the barriers during the program's implementation. The mindfulness-based coaching and small group interaction by sending empowering messages to adhere to DSM practice and answering questions were conducted to provide emotional support. | While among the control group, routine services were continuously provided by community health centers during the same period. They received the smartphone application of diabetes coaching intervention after the post-test at the end of the program implementation. |

**Appendix4 (Continued)**

| **Characteristic**  **Study ID** | **Characteristic**  **Study ID** | **Registered** | **Intervention(s)** | **Control(s)** |
| --- | --- | --- | --- | --- |
| **Stone 2010** | **Quinn 2008** | Yes | The WDS is designed to serve as a virtual coach for patients and a virtual endocrinologist for HCPs, facilitating the coordination of diabetes care among existing resources. The primary areas of focus during this 3-month trial were to test the WDS’s ability:  (1) to teach patients about dietary impacts on BG levels,  (2) to direct patients to generate higher-quality BG data, and  (3) to determine the effect of provided patient BG data, data analysis, and suggested therapy recommendations on HCP prescribing behavior.  The patient communication system used a One Touch Ultra BG meter, Bluetooth-adapted such that when the patient removed the test strip out of the BG meter, the patient’s BG value would be wirelessly, securely, and automatically sent to the patient’s cell phone. Cell phones used for the trial were either Nokia 6682 or Nokia 6680. Patient data were uploaded from the web server into the cell phone and integrated into the cell phone-based software, DiabetesManager, for personalised feedback.  Duration: 12 months. Intensity: not stated. Frequency: not stated. | At baseline, all patients completed the Summary of Diabetes Self-Care Activities (SDSCA) questionnaire and had an A1c and complete medical and demographic history obtained by the research team. Patients randomized to the control group received One Touch Ultra™  BG meters (LifeScan, Milpitas, CA) and adequate BG testing strips and lancets for the duration of the trial. They were asked to fax or call in their BG logbooks every 2 weeks to their HCPs until their BG levels were stabilized in the target ranges or until their HCPs changed testing frequency. Investigators asked treating HCPs to follow their usual standards of care for the patients’ diabetes management.  Duration: 3 months. Intensity: not stated. Frequency: up to fortnightly calls/faxes to research team. |

**Appendix4 (Continued)**

| **Characteristic**  **Study ID** | **Characteristic**  **Study ID** | **Registered** | **Intervention(s)** | **Control(s)** |
| --- | --- | --- | --- | --- |
| **Stone 2010** | **Sachmechi 2023** | No | The intervention group, using the Vivovitals diabetes platform, measured their daily fasting and postprandial BG levels. The measurements were automatically transferred from the glucometer to the Vivovitals application and then uploaded to the online web portal, where the physician reviewed all BG data. Regardless of the measured BG levels for the week, every patient in the intervention group received twice-weekly phone calls from the physician to optimize the medication regimen and provide guidance on lifestyle modification (brisk walk for at least 30 minutes 3 times weekly and a recommended diet from the American Diabetes Association consisting of 50% nonstarchy vegetables, 25% lean protein foods, and 25% carbohydrates), when necessary. If the BG level measured at any time was <70mg/dL, the physician received an alert notification and called the patient in order to provide further guidance. The patients in the intervention group also had scheduled office visits at the start and end of the trial. There was no limitation, in either group, in terms of which antidiabetic medications the patients could take. The patients enrolled in the trial were informed that only the providers involved in the study could alter their antidiabetic medication and that no other alterations should be made by their primary care physician. | The control group received usual clinical care, which consisted of office visits at the beginning and end of the trial (12-week visit), with the participants instructed to measure and log their BG levels daily. The daily BG measurements were reviewed by the physician at the patients’ 12-week visit. The participants in the control group had the option to call the physician for advice if their measured BG level was <70 mg/dL or >180 mg/dL or if they had questions regarding the medication regimen. The patients’ management was altered only if they contacted the physician. On average, the patients in the control group contacted the provider once monthly. The patients were not required to meet a diabetes educator, inperson follow-up visits were limited to 12 weeks, and adjustments to medications were not prespecified or controlled but were made on a personalized basis. Therefore, the patients in the control group received usual clinical care and not the standard of care. In addition, the providers oversaw care for all the participants and were not limited to 1 specific group. |

**Appendix4 (Continued)**

| **Characteristic**  **Study ID** | **Characteristic**  **Study ID** | **Registered** | **Intervention(s)** | **Control(s)** |
| --- | --- | --- | --- | --- |
| **Stone 2010** | **Satish 2007** | No | Subjects randomized to the experimental group received a PDA loaded with the insulin guidance software. The concept behind the Advisor software is similar to bolus calculators currently used on many insulin pumps. At baseline (visit 1), a health care provider and/or certified diabetes educator reviewed the features of the software on the PDA and loaded a subject-specific insulin dosing algorithm into the software based on the physician’s recommendations. The software program allowed the health care provider to enter demographic data such as age, height, and weight that could potentially affect the insulin sensitivity factor already programmed into the device. The program advised basal, bolus, and correction insulin dosages based on individual patients’ prescriptions in addition to being alerted for SMBG testing. Subjects in the experimental group were also asked to input their blood glucose values into the PDA via the touch screen. Subjects then received a recommended insulin dose based on their prescription, which was programmed by the health care provider. The patients were asked to either agree with the recommended insulin dose or disagree, and manually enter the insulin dose they took for a given event. All the data from the glucose meters and the PDAs were downloaded at every visit.  All subjects were asked to attend seven in-clinic visits (baseline, 2 weeks, 6 weeks, 3 months, 6 months, 9 months, and 12 months) and participate in three telephone visits (4.5 months, 7.5 months, and 9.5 months) throughout the course of the study. | Given a glucose meter and an unlimited supply of test strips for SMBG. |

**Appendix4 (Continued)**

| **Characteristic**  **Study ID** | **Characteristic**  **Study ID** | **Registered** | **Intervention(s)** | **Control(s)** |
| --- | --- | --- | --- | --- |
| **Stone 2010** | **Stone 2010** | Yes | Subjects randomized to the experimental group received a PDA loaded with Viterion 100 Monitor home telemonitoring device permits continuous home messaging with reminders and education; ongoing monitoring of SMBG, blood pressure, and weight; and daily transmission of these data to study providers via a secure network (20). Participants were instructed to transmit uploaded measurements from Viterion-compatible peripheral devices to the study nurse practitioner daily. On Monday through Friday, the nurse practitioner reviewed SMBG, blood pressure, weight, and risk stratification reports generated by the Viterion and contacted participants as necessary.  The nurse practitioner provided timely telephone follow-up, including further selfmanagement education for participants who generated “high-risk” reports based on unacceptably high or low SMBG or blood pressure readings. Medications for glycemic, blood pressure, and lipid control were adjusted by the nurse practitioner supervised by the study endocrinologist without prior approval of the PCP who was informed retrospectively of all changes. The nurse practitioner maintained records of all medication changes made in the ACMHT group. The nurse practitioner also called ACMHT participants monthly to provide individualized self-management counseling tailored to specific issues, based on the status of glucose and blood pressure control from the transmitted data.  Duration: 3 months. | Participants randomly assigned to the CC group received monthly telephone calls from the study diabetes nurse educator regarding general health conditions, status of glycemic control, blood pressure, and weight from daily logs maintained by the participants and compliance with the prescribed diabetic regimen. Issues requiring active intervention were referred to their PCP. Participants also could initiate contact with the study diabetes nurse educator to discuss concerns related to diabetes management. |
