# Supplementary material for: Impact of Digital Therapeutics for the Management of Adult Patients With Diabetes: Systematic Review and Meta-Analysis of Randomized Controlled Trials
Source: J Med Internet Res. 2025 Sep 8;27:e70428. doi: 10.2196/70428 (PMC12455173; doi:10.2196/70428)
Supplement: Multimedia Appendix 7 [file jmir_v27i1e70428_app7.docx]

**Appendix 7. Original study data**

| Study ID | Primary endpoint(s) | |  | Secondary endpoints | |
| --- | --- | --- | --- | --- | --- |
|  | Intervention group | Control group |  | Intervention group | Control group |
| Agarwal 2019 | **HbA1c (%), mean (SD)**: Unadjusted mean HbA1c values were 8.22 at 3 months, no information at 6 months, 8.89 (1.82) at baseline. The results of an ANCOVA controlling for baseline values of 120 participants (63 WLC and 57 ITG) did not show evidence of impact on HbA1c levels at 3 months for those in the intervention group. Using a paired t test, did not show a statistically significant difference in HbA1C levels between 3 and 6 months. Each additional day of app use corresponded with a 0.016-point decrease in participants’ 3month HbA1c levels (95% CI −0.03 to −0.003) | **HbA1c (%), mean (SD)**: Unadjusted mean HbA1c values were 8.41, no information at 6 months, 9.03 (1.53) at baseline |  | **Patient-reported diabetes self-care behaviors** (measured by PAID and Summary of Diabetes Self-Care Activities-6): (1) PAID: 29.6 ± 22.9 at baseline, 30.7±24.7 at 3 months; (2) SDSCA – General Diet: 4.16 ± 2.04 at baseline, 4.24 ± 1.91 at 3 months; (3) SDSCA – Specific Die: 4.38 ± 1.69 at baseline, 4.24 ± 1.51 at 3 months; (4) SDSCA – Exercise: 2.93 ± 2.33 at baseline, 3.15 ± 2.31 at 3 months; (5) SDSCA – Blood Glucose: 4.69 ± 2.53 at baseline, 3.99 ± 2.51 at 3 months; (6) SDSCA – Foot Care: 2.52 ± 2.67 at baseline, 2.46 ± 2.47 at 3 months; (7) SDSCA – of Cigarettes: 2.49 ± 7.46 at baseline, 1.61 ± 5.13 at 3 months | **Patient-reported diabetes self-care behaviors** (measured by PAID and Summary of Diabetes Self-Care Activities-6): (1) PAID: 25.9 ± 20.1 at baseline, 29.9 ± 20.7 at 3 months; (2) SDSCA – General Diet: 4.17 ± 2.19 at baseline, 4.05 ± 2.29 at 3 months; (3) SDSCA – Specific Diet: 4.54 ± 1.51 at baseline, 4.41 ± 1.68 at 3 months; (4) SDSCA – Exercise: 2.75 ± 2.20 at baseline, 2.86 ± 2.31 at 3 months; (5) SDSCA – Blood Glucose: 4.88 ± 2.40 at baseline, 4.18 ± 2.55 at 3 months; (6) SDSCA – Foot Care: 3.26 ± 2.47 at baseline, 3.16 ± 2.58 at 3 months; (7) SDSCA - of Cigarettes: 1.22 ± 4.83 at baseline, 1.27 ± 4.77 at 3 months |
|  |  |  |  | **General health status** (measured by EuroQol-5D): (1) EQ5D VAS: 64.6 ± 21.6 at baseline, 70.5 ± 20.8 at 3 months; (2) Eq5D Index: 0.77 ± 0.19 at baseline, 0.77 ± 0.18 at 3 months | **General health status** (measured by EuroQol-5D): (1) EQ5D VAS: 67.2 ± 16.8 at baseline, 68.3 ± 18.6 at 3 months; (2) Eq5D Index: 0.81 ± 0.15 at baseline, 0.81 ± 0.14 at 3 months |

| Study ID | Primary endpoint(s) | |  | Secondary endpoints | |
| --- | --- | --- | --- | --- | --- |
|  | Intervention group | Control group |  | Intervention group | Control group |
| Benhamou 2019 | **Percentage of time spent in the 3.9 ~ 10.0 mmol/L (70 ~ 180 mg/dL) glucose target range based on continuous glucose monitoring**: (1) 3.9 ~ 10.0 mmol/L: 68.5%; (2) 4.4 ~ 7.8 mmol/L: 39.3%; (3) > 10.0 mmol/L: 29.5%; (4) > 13.9 mmol/L: 7.4%; (5) > 16.7 mmol/L: 2.4%; (6) < 3.9 mmol/L: 2.0%; (7) < 3.3 mmol/L: 0.8%; (8) < 2.8 mmol/L: 0.2%. | **Percentage of time spent in the 3.9 ~ 10.0 mmol/L (70 ~ 180 mg/dL) glucose target range based on continuous glucose monitoring**: (1) 3.9 ~ 10.0 mmol/L: 59.4%; (2) 4.4 ~ 7.8 mmol/L: 33.5%; (3) > 10.0 mmol/L: 36.3%; (4) > 13.9 mmol/L: 11.7%; (5) > 16.7 mmol/L: 4.3%; (6) < 3.9 mmol/L: 4.3%; (7) < 3.3 mmol/L: 2.0%; (8) <2.8 mmol/L: 0.7% |  | **Percentage of time sensor glucose concentration within the optimal target range (4.4 ~ 7.8 mmol/L)**: 39.3% | **Percentage of time sensor glucose concentration within the optimal target range (4.4 ~ 7.8 mmol/L)**: 33.5% |
|  | **HbA1c (%), mean (SD)**：7.6 (0.9) at baseline, decreased 0.29 (0.6) at 12 weeks. |  |  | **HbA1c change from baseline (%), mean (SD)**: decreased 0.29 (0.6) at 12 weeks | **HbA1c change from baseline (%), mean (SD)**: decreased 0.14 (0.6) at 12 weeks |
|  |  |  |  | **Glucose concentration (mmol/L), mean (SD)**: 8.7 (0.8) | **Glucose concentration (mmol/L), mean (SD)**: 9.1 (0.8) |
|  |  |  |  | **Coefficient of variation of glucose (%), mean (SD)**: 31.0 (3.9) | **Coefficient of variation of glucose (%), mean (SD)**: 33.3 (3.9) |
|  |  |  |  | **Low blood glucose index, mean (SD)**: 0.6 (0.8) | **Low blood glucose index, mean (SD)**: 1.1 (0.8) |
|  |  |  |  | **High blood glucose index, mean (SD)**: 6.7 (2.4) | **High blood glucose index, mean (SD)**: 8.4 (2.4) |
|  |  |  |  | **Blood glucose risk index, mean (SD)**: 7.3 (2.4) | **Blood glucose risk index, mean (SD)**: 9.5 (2.4) |
|  |  |  |  | **Diabetic ketoacidosis**: 0 | **Diabetic ketoacidosis**: 0 |
|  |  |  |  | **Severe hyperglycaemia (capillary blood glucose > 20 mmol/L),** : 9 events | **Severe hyperglycaemia (capillary blood glucose >20 mmol/L), mean (SD)**: 0 |
|  |  |  |  | **Severe hypoglycaemia (intervention of a third party for correction of hypoglycaemia)**: 5 events | **Severe hypoglycaemia (intervention of a third party for correction of hypoglycaemia),**: 3 events |

| Study ID | Primary endpoint(s) | |  | Secondary endpoints | |
| --- | --- | --- | --- | --- | --- |
|  | Intervention group | Control group |  | Intervention group | Control group |
| Bergenstal 2019 | **HbA1c (%), mean (SD)**: 8.7 (0.8) at baseline, the mean decrease in HbA1c from baseline to 6 months was 1.0 (1.0) in the intervention group | **HbA1c (%), mean (SD):** 8.5 (0.8) at baseline, the mean decrease in HbA1c from baseline to 6 months was 0.3 (0.9) in the control group (p < 0.0001 between groups; effect size 0.7%, 95% CI 0.4 to 1.0) |  | **Percentage of HbA1c of less than 7% (53 mmol/mol)**: 0 at baseline, 20 participants account for 22% at 6 months | **Percentage of HbA1c of less than 7% (53 mmol/mol)**: 0 at baseline, 4 participants account for 5% at 6 months |
|  |  |  |  | **Percentage of HbA1c of less than 8% (64 mmol/mol)**: 21 participants account for 23% at baseline, 58 participants account for 62% at 6 months | **Percentage of HbA1c of less than 8% (64 mmol/mol)**: 28 participants account for 32% at baseline, 29 participants account for 33% at 6 months |
|  |  |  |  | **Percentage of HbA1c of more than 9·0% (75 mmol/mol)**: 24 participants account for 26% at baseline, 10 participants account for 11% at 6 months | **Percentage of HbA1c of more than 9·0% (75 mmol/mol)**: 20 participants account for 23% at baseline, 7 participants account for 8% at 6 months |
| Bretschneider 2022 | **HbA1c (%)**: 7.9 ± 1.0 at baseline, 6.9 ± 0.9 at 3 months, decreased on average by 0.9 ± 1.1% (p<0.001) | **HbA1c (%)**: 8.2 ± 1.3 at baseline, 7.9 ± 1.0 at 3 months, decreased on average by 0.3 ± 1.1% (p=0.27) |  | **Fasting glucose (mmol/L)**: 7.4 ± 1.4 at baseline, decreased by 0.6 ± 1.3 (p=0.01). | No information |
|  |  |  |  | **Body weight(kg)**: 105.2 ± 18.5 at baseline, decreased by 4.3 ± 4.kg or 4% of the baseline bodyweight (p<0.001) |  |
|  |  |  |  | **Waist circumference (cm)**: 121.1 ± 16.5 at baseline, decreased by 5.7 ± 15 (p=0.03) |  |

| Study ID | Primary endpoint(s) | |  | Secondary endpoints | |
| --- | --- | --- | --- | --- | --- |
|  | Intervention group | Control group |  | Intervention group | Control group |
| Charpentier 2011 | **I1: HbA1c (%), mean (SD)**: 9.19 (1.14) at baseline, 8.63 (1.07) at 6 months, P = 0.022 | **HbA1c (%), mean (SD)**: 8.91 (0.90) at baseline, 9.10 (1.16) at 6 months |  | **I1: Proportion of patients reaching the HbA1c target of below 7.5%**: 17% (n = 10) | **Proportion of patients reaching the HbA1c target of below 7.5%**: 1.6% (n = 1) |
|  | **I2: HbA1c (%), mean (SD)**: 9.11 (1.14) at baseline, 8.41 (1.04) 6 months, P = 0.0019 |  |  | **I1: Change in SMPG frequency**: a slightly higher daily for the 14 days before the end point visit (3.57 ± 1.35, P=0.036), but with similar increases in the three groups and without difference in SMPG frequency between groups | **Amount of time spent by investigators conducting face-to-face visits or teleconsultations**: 70631 min |
|  |  |  |  | **I1: Satisfaction assessed by Diabetes Health Profile and Diabetes QOL questionnaires**: did not differ between groups |  |
|  |  |  |  | **I1: Amount of time spent by investigators conducting face-to-face visits or teleconsultations**: 70622 min |  |
|  |  |  |  | **I2: Proportion of patients reaching the HbA1c target of below 7.5%**: 6.7% (n = 4) **I2: Change in SMPG frequency**: a negative correlation between HbA1c and actual SMPG frequency for I2 participants (r = 20.34; P = 0.018). |  |
|  |  |  |  | **I2: Amount of time spent by investigators conducting face-to-face visits or teleconsultations**: 72630 min |  |

| Study ID | Primary endpoint(s) | |  | Secondary endpoints | |
| --- | --- | --- | --- | --- | --- |
|  | Intervention group | Control group |  | Intervention group | Control group |
| Franc 2019 | **HbA1c (%):** HbA1c levels decreased significantly more in patients from the investigational arms (I1: 7.42% ± 0.91% and I2: 7.47% ± 0.90%) than in the control arm (C: 7.96% ± 0.88%, P < 0.002). HbA1c decreases from baseline were also significantly higher in I1 (−1.44%) and I2 (−1.48%) arms compared with the control arm,C (−0.92%, P < 0.002) | |  | **Percentage of patients reaching HbA1c < 7.0%**: (I1: 32.8% and I2: 29.8%) achieved in twice as many patients as in the control arm (C: 12.5%, P < 0.02) | |
|  |  | |  | **Percentage of patients reaching FBG between 73 and 108 mg/dL**: (I1: 82.5% and I2: 82.7%)achieved in twice as many patients as in the control arm (C: 41.8%, P < 0.001) | |
|  |  | |  | **FBG (average of the last four days)**: (I1: 115 ± 26 mg/dL and I2: 112 ± 25 mg/dL) significantly lower compared with the control arm (C: 132 ± 24 mg/dL, P < 0.002) | |
|  |  | |  | **Pre- and postprandial BG**: significantly lower in I1 and I2 compared with C | |

| Study ID | Primary endpoint(s) | |  | Secondary endpoints | |
| --- | --- | --- | --- | --- | --- |
|  | Intervention group | Control group |  | Intervention group | Control group |
| Franc 2020 | **I1: HbA1c (%), mean (SD)**: 9.1 (1.1) at baseline, decreased by 0.26% at 12 months, P=0.022 | **HbA1c (%), mean (SD)**: 9.1 (1.0) at baseline, decreased by 0.20% at 12 months |  | **Predictive factors of glycemic control**: Significant decreases in HbA1c levels with respect to the control arm were observed in patients with baseline HbA1c ≤ 9.5% (P < 0.029, N=171 for I1; and P = 0.005, N = 157 for I2) but not in patients with baseline HbA1c > 9.5% (P = 0.826, N = 60 for I1; and P = 0.072, N = 56 for I2) | |
|  | **I1: DS usage during the first month**: at least once a day was found for 30% ~ 40% of patients, while only 20% ~ 30% of patients used DIABEO® at least twice a day |  |  | **Predictive factors of DS usage**: DIABEO® usage was significantly lower in patients with baseline HbA1c > 9.5% (P = 0.036 for I1 and P = 0.005 for I2) or aged < 25 years old (P = 0.002 for arm 2 and P = 0.009 for arm 3). Patients from arm 2 living in rural areas used DIABEO® (at least twice daily) more than patients living in urban areas (23.5% versus 10.9%, P = 0.030) (this was not the case for patients from I2). | |
|  | **I2: HbA1c (%), mean (SD)**: 9.1 (0.9) at baseline, decreased by 0.34% 12 months, P=0.0019 |  |  | **Hypoglycemia**: No significant differences between 3 groups | |
|  | **I2: DS usage during the first month**: at least once a day was found for 50% ~ 60% of patients, while only 40% ~ 50% of patients used DIABEO® at least twice a day |  |  | **Quality of life**: No significant differences between 3 groups | |
| Guo 2021 | **HbA1c (%), mean (SD)**:8.97 (2.12) at baseline, 7.81 (2.04) at 4 weeks, P = 0.014 | **HbA1c (%), mean (SD)**: 9.14 (1.96) at baseline, 9.05 (1.88) at 4 weeks |  | **Quality of life (DSQL), mean (SD)**: 42.34 (10.01) at 4 weeks | **Quality of life (DSQL), mean (SD)**: 53.28 (10.55) at 4 weeks |
|  | **BMI (kg/m^2^), mean (SD)**: 24.00 (3.13) at baseline, 22.12(2.35) at 4 weeks. | **BMI (kg/m^2^), mean (SD)**: 24.32(3.14)at baseline, 24.08(4.18) at 4 weeks |  | **Self-management ability, mean (SD)**: 55.69 (6.74) at 4 weeks, P < 0.001 | **Self-management ability, mean (SD)**: 39.69 (8.32) at 4 weeks |
|  | **FBG (mmol/L), mean (SD)**: 7.81 (1.77) at baseline, 6.59 (1.03) at 4 weeks, P = 0.001 | **FBG (mmol/L), mean (SD)**: 8.11(1.41) at baseline, 7.84(1.83) at 4 weeks |  |  |  |
|  | **Postprandial two-hour blood glucose (2hPG) (mmol/L), mean (SD)**: 9.51 (1.42)at baseline, 8.46 (1.28) at 4 weeks, P = 0.033 | **Postprandial two-hour blood glucose (2hPG) (mmol/L), mean (SD)**: 9.62 (1.63) at baseline, 9.18 (1.36) at 4 weeks |  |  |  |

| Study ID | Primary endpoint(s) | |  | Secondary endpoints | |
| --- | --- | --- | --- | --- | --- |
|  | Intervention group | Control group |  | Intervention group | Control group |
| Hsia 2022 | **HbA1c (%), mean (SD)**: 8.2 (0.1) at baseline, decreased 0.28% (95% CI -0.41, -0.15) at 3 months | **HbA1c (%), mean (SD)**: 8.1 (0.1) at baseline, increased 0.11% (95% CI -0.02, 0.23) at 3 months |  | **Adverse events**: 68 (21%) of subjects | **Adverse events**: 76 (22%) of subjects |
|  |  |  |  | **Weight (kg), mean (SD)**: 99 (20) at baseline, decreased 1.5 (6.3) at 3 months | **Weight (kg), mean (SD)**: 101 (24) at baseline, decreased 1.0 (5.5) at3 months. |
|  |  |  |  | **SBP (mmHg), mean (SD)**: 127 (15) at baseline, decreased 3.0 (15.4) at 3 months | **SBP (mmHg), mean (SD)**: 126 (14) at baseline, decreased 1.0 (5.5) at 3 months |
|  |  |  |  | **DBP (mmHg), mean (SD)**: 78 (10) at baseline, decreased 1.5 (9.7) at 3 months | **DBP (mmHg), mean (SD)**: 78 (9) at baseline, decreased 1.0 (9.8) at3 months |
|  |  |  |  | **Total Cholesterol (mmol/L), mean (SD)**: 4.4 (1.1) at baseline, decreased 0.13 (0.74) at 3 months | **Total Cholesterol (mmol/L), mean (SD)**: 4.4 (1.2) at baseline, decreased 0.08 (0.87) at3 months |
|  |  |  |  | **HDL (mmol/L), mean (SD)**: 1.2 (0.3) at baseline, decreased 0.01 (0.15) at 3 months. | **HDL (mmol/L), mean (SD)**: 1.2 (0.3) at baseline, increased 0.02 (0.15) at 3 months |
|  |  |  |  | **LDL (mmol/L), mean (SD)**: 2.4 (0.9) at baseline, decreased 0.11 (0.58) at 3 months | **LDL (mmol/L), mean (SD)**: 2.4 (0.9) at baseline, decreased 0.06 (0.70) at 3 months |
|  |  |  |  | **Triglycerides (mmol/L), mean (SD)**: 2.2 (1.5) at baseline, decreased 0.15 (0.90) at 3 months | **Triglycerides (mmol/L), mean (SD)**: 2.3 (4.3) at baseline, decreased 0.05 (1.31) at 3 months |
|  |  |  |  |  | **FBG (mmol/L), mean (SD)**: 9.3 (3.0) at baseline, decreased 0.07 (3.28) at 3 months |

| Study ID | Primary endpoint(s) | |  | Secondary endpoints | |
| --- | --- | --- | --- | --- | --- |
|  | Intervention group | Control group |  | Intervention group | Control group |
| Hsu 2016 | **HbA1c (%), mean (SD)**: 10.8 at baseline, 7.7 (1.6) at 12 weeks, achieved a mean HbA1c decrease of 3.2 ± 1.5% (P < 0.0001) | **HbA1c (%), mean (SD)**: 10.9 at baseline, 8.9 (2.2) at 12 weeks, decreased by 2.0 ± 2.0% (P = 0.0003) |  | **Diabetes Treatment Satisfaction Questionnaire (DTSQ), mean (SD)**: 31.9 (10.1) points at baseline, 42.0 (3.8) points at 12 weeks, P=0.001 | **Diabetes Treatment Satisfaction Questionnaire (DTSQ), mean (SD)**: 34.3 (8.5) points at baseline, 36.4 (8.9) points at 12 weeks, P=0.1 |
|  |  |  |  | **Time HCPs and subjects spent on managing the insulin titration (minutes)**: 22.5 | **Time HCPs and subjects spent on managing the insulin titration (minutes)**: 68.8 |
|  |  |  |  | **Frequency for hypoglycemia**: 4 participants | **Frequency for hypoglycemia**: 2 participants |
|  |  |  |  | **the percentage reaching the glycemic target of A1c ≤ 7%**：There was no statistical difference between 2 groups | |
| Jafar 2023 | **HbA1c (%)**: Post-HbA1c level on both intervention (9.06 ± 2.05) and control groups (8.64 ± 1.63) are lower than pre-HbA1c level on both intervention (9.56 ± 1.95) and control groups (8.66 ± 1.69), but the decrease was not significant (p > 0.05) | |  | **Diabetes self-management knowledge**: increased in the postintervention period of the intervention group (14.97 ± 3.27 ~ 15 ± 3.80, p=0.0001), with an average increase of 4.10 ± 3.49 points. A significant difference in the average points increase between the two groups (4.10 ± 3.49 ~ 0.64 ± 3.96, p = 0.001) | |
|  | **HbA1c reduction**: Difference in the number of HbA1c level reductions between the intervention (−0.503 ± 1.60) and control (−0.018 ± 1.81) groups was not statistically significant (p > 0.271) | |  | **Quality of life**: A comparison of the QOL scores before and after the intervention showed a significant difference (54.34 ± 6.44 ~ 60.28 ± 6.31, p = 0.006). When comparing the change in QOL scores between the intervention group (5.93 ± 6.38) and control group (−1.82 ± 5.19), a significant difference was found (p = 0.0001) | |

| Study ID | Primary endpoint(s) | |  | Secondary endpoints | |
| --- | --- | --- | --- | --- | --- |
|  | Intervention group | Control group |  | Intervention group | Control group |
| Lee 2018 | **HbA1c (%), mean (SD)**: 8.1 (1.5) at baseline, 7.5 (1.1) at 6 months，p < 0.001 | **HbA1c (%), mean (SD)**: 8.0 (1.2) at baseline, 7.9 (1.5) at 6 months, p = 0.46 |  | **BMI (kg/m^2^)**: 26.1 ± 3.3 at baseline, 25.7 ± 3.4 at 6 months, p = 0.01 | **BMI (kg/m^2^)**: 26.3 ± 3.2 at baseline, 25.7 ± 3.3 at 6 months, p < 0.001. |
|  |  |  |  | **HDL (mg/dL)**: 47.3 ± 11.2 at baseline, 48.7 ± 10.4 at 6 months, p = 0.11 | **HDL (mg/dL)**: 48.0 ± 14.9 at baseline, 48.7 ± 12.2 at 6 months, p = 0.62 |
|  |  |  |  | **LDL (mg/dL)**: 93.2 ± 30.0 at baseline, 94.2 ± 33.8 at 6 months, p = 0.75 | **LDL (mg/dL)**: 87.1 ± 38.1 at baseline, 98.3 ± 41.0 at 6 months, p = 0.01 |
|  |  |  |  | **SBP (mmHg)**: 137.1 ± 15.9 at baseline, 120.3 ± 10.4 at 6 months, p < 0.001 | **SBP (mmHg)**: 138.8 ± 16.1 at baseline, 119.7 ± 11.2 at 6 months, p < 0.001 |
|  |  |  |  | **DBP (mmHg)**: 87.0 ± 10.4 at baseline, 78.6 ± 7.9 at 6 months, p < 0.001 | **DBP (mmHg)**: 86.6 ± 9.6 at baseline, 79.2 ± 7.4 at 6 months, p < 0.001 |
|  |  |  |  | **SDSCA scores**: (1) Diet total (times/week): 2.7 ± 1.5 at baseline, 2.9 ± 1.1 at 6 months; (2) Exercise (times/week): 2.9 ± 1.8 at baseline, 3.5 ± 1.8 at 6 months; (3) SMBG (times/week): 1.7 ± 2.2 at baseline, 3.5 ± 2.3 at 6 months; (4) Foot care (times/week): 1.5 ± 1.8 at baseline, 3.1 ± 2.5 at 6 months; (5) Smoking (times/week): 2.1 ± 3.2 at baseline, 1.8 ± 3.0 at 6 months; (6) Number of cigarettes /day: 4.5 ± 8.0 at baseline, 3.3 ± 6.8 at 6 months | **SDSCA scores**: (1) Diet Total (times/week): 2.6 ± 1.3 at baseline, 2.8 ± 1.2 at 6 months; (2) Exercise (times/week): 3.0 ± 1.6 at baseline, 2.9 ± 1.4 at 6 months; (3) SMBG (times/week): 1.6 ± 2.2 at baseline, 1.9 ± 2.2 at 6 months; (4) Foot care (times/week): 1.5 ± 2.0 at baseline, 2.5 ± 2.1 at 6 months; (5) Smoking (times/week): 1.8 ± 3.0 at baseline, 1.8 ± 3.0 at 6 months; (6) Number of cigarettes /day: 3.5 ± 7.2 at baseline, 2.3 ± 5.7 at 6 months |
|  |  |  |  | **Total Appraisal of Diabetes Scale (ADS) scores**: 18.6 ± 4.3 at baseline, 17.9 ± 3.3 at 6 months | **Total ADS scores**: 18.8 ± 3.3 at baseline, 17.9 ± 3.3 at 6 months |

| Study ID | Primary endpoint(s) | |  | Secondary endpoints | |
| --- | --- | --- | --- | --- | --- |
|  | Intervention group | Control group |  | Intervention group | Control group |
| Lim 2022 | **Weight loss, mean (SD)**: 82.7 (15.2) kg at baseline, decreased 4.2 (4.5) kg at 6 months | **Weight loss, mean (SD)**: 81.3 (12.5) kg at baseline, decreased 1.3 (3.9) kg at 6 months |  | **HbA1c (%), mean (SD)**: 5.94 (0.48) at baseline, decreased 0.22 (0.33) at 6 months | **HbA1c (%), mean (SD)**: 6.06 (0.50) at baseline, decreased 0.06 (0.26) at 6 months |
|  |  |  |  | **FBG (mmol/L), mean (SD)**: 6.22 (0.85) at baseline, decreased 0.37 (0.88) at 6 months | **FBG (mmol/L), mean (SD)**: 6.24 (0.79) at baseline, increased 0.01 (0.74) at 6 months |
|  |  |  |  | **SBP (mmHg), mean (SD)**: 137.3 (18.1) at baseline, decreased 6.1 (14.2) at 6 months | **SBP (mmHg), mean (SD)**: 135.6 (16.5) at baseline, decreased 2.5 (17) at 6 months |
|  |  |  |  | **DBP (mmHg), mean (SD)**: 83.0 (11.7) at baseline, decreased 3.2 (10.4) at 6 months | **DBP (mmHg), mean (SD)**: 82.2 (10.5) at baseline, decreased 1.9 (9.6) at 6 months |
|  |  |  |  | **Total cholesterol (mmol/L), mean (SD)**: 5.16 (0.96) at baseline, decreased 0.29 (0.89) at 6 months | **Total cholesterol (mmol/L), mean (SD)**: 4.92 (1.03) at baseline, decreased 0.17 (0.99) at 6 months |
|  |  |  |  | **HDL (mmol/L), mean (SD)**: 1.28 (0.25) at baseline, increased 0.09 (0.22) at 6 months | **HDL (mmol/L), mean (SD)**: 1.32 (0.29) at baseline, decreased 0 (0.23) at 6 months |
|  |  |  |  | **LDL (mmol/L), mean (SD)**: 3.13 (0.85) at baseline, decreased at 0.31 (0.84) 6 months | **LDL (mmol/L), mean (SD)**: 2.84 (0.92) at baseline, decreased 0.13 (0.95) at 6 months |
|  |  |  |  | **Triglycerides (mmol/L), mean (SD)**: 1.70 (0.83) at baseline, decreased 0.19 (0.77) at 6 months | **Triglycerides (mmol/L), mean (SD)**: 1.76 (1.33) at baseline, decreased 0.11 (1.58) at 6 months |
|  |  |  |  | **Physical activity (minutes/week), mean (SD)**: 102.0 (141.5) at baseline, increased 44.2 (144.8) at 6 months | **Physical activity (minutes/week), mean (SD)**: 89.2 (103.0) at baseline, increased 11.2 (123.4) at 6 months |
|  |  |  |  | **Proportion of participants with ≥ 5% weight loss (%)**: 33 (45.8) | **Proportion of participants with ≥ 5% weight loss (%)**: 9 (11.8) |

| Study ID | Primary endpoint(s) | |  | Secondary endpoints | |
| --- | --- | --- | --- | --- | --- |
|  | Intervention group | Control group |  | Intervention group | Control group |
| Moravcová 2022 | **HbA1c (%), mean (SD)**: 7.2 (1.3) at baseline, 6.2 (0.6) at 3 months | **HbA1c (%), mean (SD)**: 6.7 (1.9) at baseline, 6.3 (1.4) at 3 months |  | **HOMA-IR (µkat/L), mean (SD)**: 12.6 (10.3) at baseline, 3.5 (2.2) at 3 months | **HOMA-IR (µkat/L), mean (SD)**: 4.8 (2.1) at baseline, 6.8 (4.5) at 3 months |
|  |  |  |  | **FG (mmol/L), mean (SD)**: 9.3 (3.7) at baseline, 7.1 (1.1) at 3 months | **FG (mmol/L), mean (SD)**: 7.9 (2.8) at baseline, 7.8 (2.8) at 3 months |
| Pamungkas 2022 | **HbA1c (%), mean (SD)**: 8.04 (1.96) at baseline, 6.44 (1.14) at 12 weeks. | **HbA1c (%), mean (SD)**: 8.55 (2.95) at baseline, 8.24 (2.60) at 12 weeks |  | **SBP(mmHg), mean (SD)**: 128.67 (13.83) at baseline, 120.00 (11.14) at 12 weeks | **SBP(mmHg), mean (SD)**: 128.33 (18.21) at baseline, 8.24 (2.61) at 12 weeks |
|  | **BMI (kg/m^2^), mean (SD)**: 23.7 (3.52) at baseline, 23.58 (2.80) at 12 weeks | **BMI (kg/m^2^), mean (SD)**: 24.32 (3.51) at baseline, 24.28 (2.69) at 12 weeks |  | **DBP (mmHg), mean (SD)**: 83.33 (7.11) at baseline, 72.50 (8.66) at 12 weeks | **DBP (mmHg), mean (SD)**: 82.00 (8.87) at baseline, 78.00 (10.95) at 12 weeks |
|  |  |  |  | **HDL (mmol/L), mean (SD)**：65.17 (14.41) at baseline, 91.8 (20.72) at 12 weeks | **HDL (mmol/L), mean (SD)**：65.47 (23.82) at baseline, 61.57 (19.35) at 12 weeks |
|  |  |  |  | **LDL(mmol/L), mean (SD)**：117.63 (49.61) at baseline, 89.10 (14.91) at 12 weeks | **LDL(mmol/L), mean (SD)**：107.50 (37.25) at baseline, 109.91 (35.66) at 12 weeks |
|  |  |  |  | **Indonesian version of diabetes self-management (DSM) questionnaire, mean (SD)**: (1) Dietary control: 3.97 (1.50) at baseline, 8.83 (1.80) at 12 weeks; (2) Physical exercise: 3.43 (2.06) at baseline, 6.87 (1.36) at 12 weeks; (3) SMBG: 4.53 (1.78) at baseline, 10.23 (1.48) at 12 weeks; (4) Medication adherence: 2.57 (1.38) at baseline, 4.97 (1.0) at 12 weeks; (5) Screening of diabetes complication: 1.90 (2.07) at baseline, 6.27 (1.26) at 12 weeks | **Indonesian version of diabetes self-management (DSM) questionnaire, mean (SD)**: (1) Dietary control: 4.37 (1.27) at baseline, 5.17 (1.84) at 12 weeks; (2) Physical exercise: 3.23 (1.46) at baseline, 3.77 (1.25) at 12 weeks; (3) SMBG: 4.07 (1.57) at baseline, 7.40 (1.35) at 12 weeks; (4) Medication adherence: 3.03 (1.79) at baseline, 3.10 (0.61) at 12 weeks; (5) Screening of diabetes complication: 1.97 (1.81) at baseline, 2.13 (1.20) at 12 weeks |

| Study ID | Primary endpoint(s) | |  | Secondary endpoints | |
| --- | --- | --- | --- | --- | --- |
|  | Intervention group | Control group |  | Intervention group | Control group |
| Quinn 2008 | **HbA1c (%), mean**: 9.51 at Baseline, 7.48 at 3 months, P = 0.04 | **HbA1c (%), mean**: 9.05 at baseline, 8.37 at 3 months, P = 0.04 |  | **Knowledge:** **Improved knowledge of food choices (self-reported)**: 90.91% at 3 months, no baseline | **Knowledge:** **Improved knowledge of food choices (self-reported)**: 50% at 3 months, no baseline |
|  | **Patient confident about DM control (self-reported)**: 100% at 3 months, no baseline | **Patient confident about DM control (self-reported)**: 75% at 3 months, no baseline |  | **Diet: SDSCA scores - Diet:** baseline 3.15, 3 months 5.5 | **Diet: SDSCA scores - Diet:** baseline 3.15, 3 months 3.86 |
|  |  |  |  | **Exercise: SDSCA scores - Exercise:** baseline 2.08, 3 months 2.92 | **Exercise: SDSCA scores - Exercise:** baseline 1.23, 3 months 1.57 |
|  |  |  |  | **Adherence: SDSCA scores - Medications:** baseline 5.92, 3 months 6.64 | **Adherence: SDSCA scores - Medications:** baseline 6.3, 3 months 6.75 |
|  |  |  |  | **Depression: New diagnosis of depression at 3 months**: 9.09% | **Depression: New diagnosis of depression at 3 months**: 20% |
|  |  |  |  | **Medication intensified**: 84.62% | **Medication intensified**: 23.08% |
|  |  |  |  | **Medication errors identified**: 53.38% | **Medication errors identified**: 0% |
| Sachmechi 2023 | **HbA1c (%), mean (SD)**: The average was 9.5 (1.7) at baseline. The median was 9.1 at baseline, 8.0 at 12 weeks. There were statistically significant reductions in the HbA1c values in the intervention group between the baseline and after 12 weeks (P ≤ 0.001) | **HbA1c (%), mean (SD)**: The average was 9.6 (1.4) at baseline. The median was 9.1 at baseline, 8.0 at 12 weeks. No statistical significance was found in the HbA1c level in the control group between the baseline and after 12 weeks (P = 0.213) |  | No information | |

| Study ID | Primary endpoint(s) | |  | Secondary endpoints | |
| --- | --- | --- | --- | --- | --- |
|  | Intervention group | Control group |  | Intervention group | Control group |
| Satish 2007 | **HbA1c (%), mean (SD)**: The intervention group maintained a 0.64% reduction. It was significantly lower in the intervention group from 3 to 12 months compared to the control group | **HbA1c (%), mean (SD)**: There was no significant difference in subjects reaching target A1c values between the control and intervention groups at 12 months |  | **Hypoglycemia, n**: 26, the intervention group had a significant increase | **Hypoglycemia, n**: 8 |
|  | **Glucose target ranges:** At 3, 6, and 9 months the mean percentage of glucose readings WTR was significantly greater in the intervention group than in the control group. The percentage of BTR was also higher in the intervention group at 3, 6, 9, and 12 months than in the control group | **Glucose target ranges:** ATR was significantly higher in the control group at 3, 6, 9, and 12 months |  | **Insulin dose**: 36 ± 2 units at baseline, there was no difference at any other time | **Insulin dose**: 43 ± 2 units at baseline |
|  |  |  |  | **Body weight**: gain from baseline to 12 months of 3.44 ± 10.59 pounds | **Body weight**: gain from baseline to 12 months of 5.30 ± 7.81 pounds |
|  |  |  |  | **Blood glucose testing**: Subjects in the intervention group showed a significant increase in number of tests per subject per day as compared to the control group at 6 weeks, 3, 6, 9, and 12 months | |

| Study ID | Primary endpoint(s) | |  | Secondary endpoints | |
| --- | --- | --- | --- | --- | --- |
|  | Intervention group | Control group |  | Intervention group | Control group |
| Stone 2010 | **HbA1c (%), mean (SD)**: 9.6 (1.6) at baseline, 7.9 (1.2) at 6months | **HbA1c (%), mean (SD)**: 9.4 (1.4) at baseline, 8.6 (1.3) at 6months |  | **Self-monitored blood glucose (SMBG)**: 7 participants (10.9%) never transmitted any SMBG data after initial training; 9 participants (14.1%) performed SMBG on average < 1 time per day, whereas 75.0% performed SMBG between 1 and 4 times per day (average 2.3 times daily) during the period in which they transmitted measurements. Among the 57 participants who transmitted measurements, 35 (61.4%) transmitted SMBG < 50 mg/dl on at least 1 day (median 1 day) and 16 participants (28.1%) transmitted SMBG between 50 and 70 mg/dl (median 10 days). Within the intervention group, the frequency of SMBG did not correlate significantly with reduction in A1C (r = -0.11; P = 0.39) |  |
|  | **Weight (kg), mean (SD)**: 102.78 (20.59) at baseline, 104.1 (21.59) at 6 months | **Weight (kg), mean (SD)**: 101.38 (21.73) at baseline, 101.56 (22.04) at 6 months |  | **Nurse-to-participant telephone contact time:** intervention group was substantially greater than control group (1.3 vs. 0.3 h/participant/ month, respectively) | |
|  | **Systolic blood pressure (mmHg)**, **mean (SD)**: 144.8 (21.7) at baseline，132.0 (24.3) at 6months | **Systolic blood pressure (mmHg)**, **mean (SD)**: 142.3 (19.0) at baseline, 133.0 (19.0) at 6 months |  |  |  |
|  | **Diastolic blood pressure (mmHg), mean (SD)**: 79.9 (13.3) at baseline, 72.4 (14.6) at 6months | **Diastolic blood pressure (mmHg), mean (SD)**: 80.5 (10.1) at baseline, 75.9 (13.2) at 6 months |  |  |  |
|  | **Cholesterol (mg/dl), mean (SD)**: 177.3 (54.2) at baseline, 148.2 (40.2) at 6months | **Cholesterol (mg/dl), mean (SD)**: 175.6 (43.5) at baseline, 159.1 (37.2) at 6 months |  |  |  |
|  | **HDL (mg/dl), mean (SD)**: 38.4 (13.5) at baseline, 35.1 (11.3) at 6months | **HDL (mg/dl), mean (SD)**: 38.4 (13.0) at baseline, 36.4 (13.6) at 6 months |  |  |  |
|  | **LDL (mg/dl), mean (SD)**: 98.8 (36.3) at baseline, 82.3 (27.9) at 6months | **LDL (mg/dl), mean (SD)**: 101.8 (32.0) at baseline, 91.2 (30.6) at 6 months |  |  |  |
|  | **Triglycerides (mg/dl), mean (SD)**: 191.3 (133.3) at baseline, 152.4 (99.7) at 6months | **Triglycerides (mg/dl), mean (SD)**: 194.1 (160.4) at baseline, 170.7 (115.9) at 6 months |  |  |  |
