# Supplementary material for: Impact of Digital Therapeutics for the Management of Adult Patients With Diabetes: Systematic Review and Meta-Analysis of Randomized Controlled Trials
Source: J Med Internet Res. 2025 Sep 8;27:e70428. doi: 10.2196/70428 (PMC12455173; doi:10.2196/70428)

**Appendix 8. Forest plots**

**Figure 1.** Subgroup analysis of HbA1c


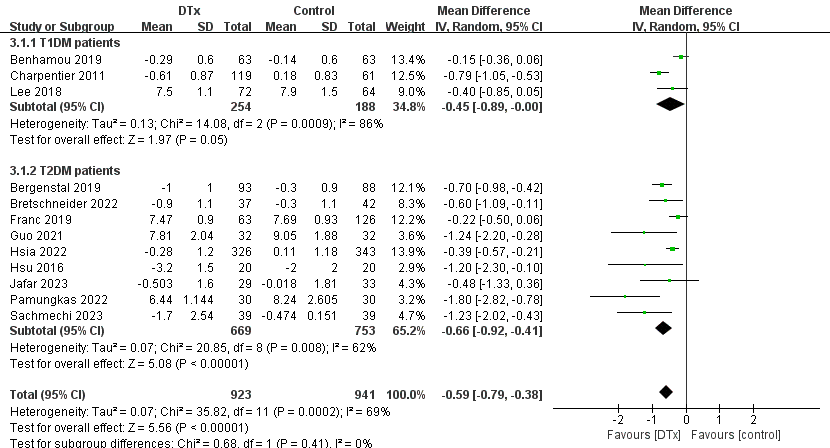


**Figure 2.** Forest plot showing effectiveness of DTx for diabetes management in improving BMI


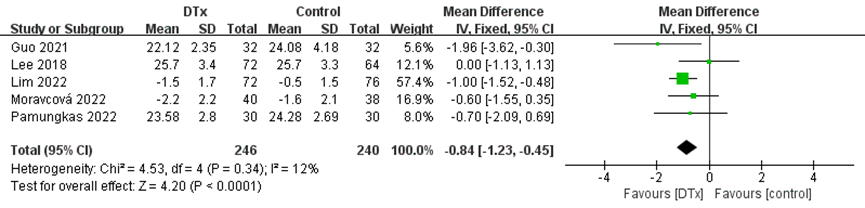


**Figure 3.** Subgroup analysis of BMI


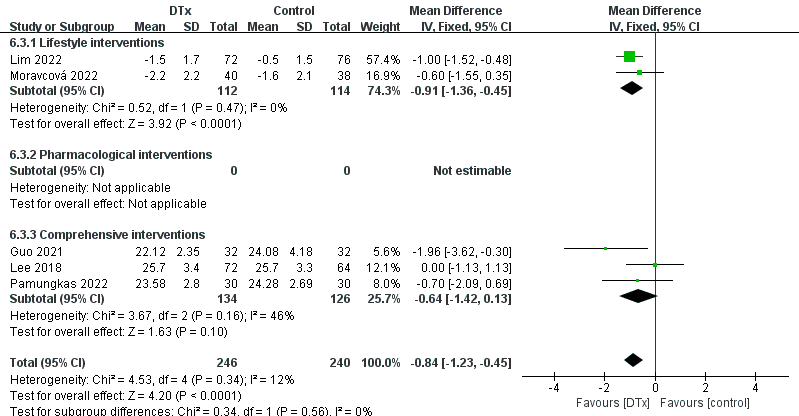


**Figure 4.** Forest plot showing effectiveness of DTx for diabetes management in improving weight


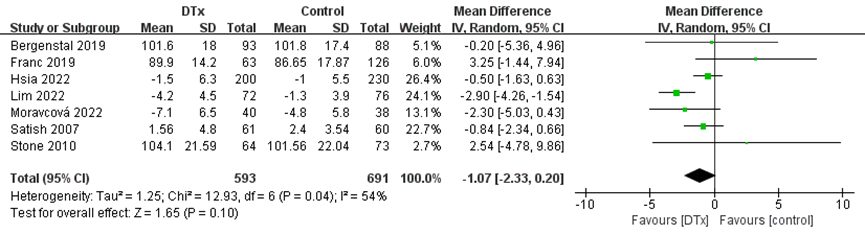


**Figure 5.** Subgroup analysis of Weight


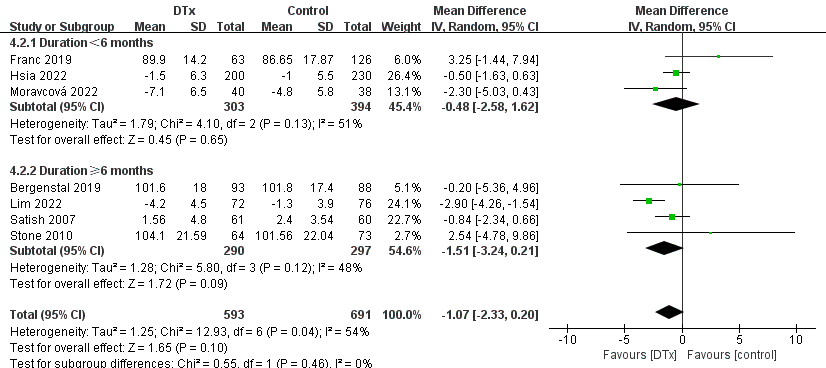


**Figure 6.** Forest plot showing effectiveness of DTx for diabetes management in improving FBG


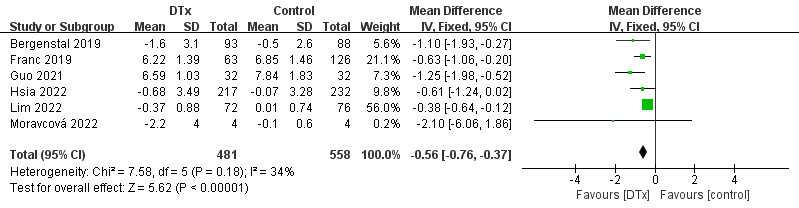


**Figure 7.** Forest plot showing effectiveness of DTx for diabetes management in improving SBP


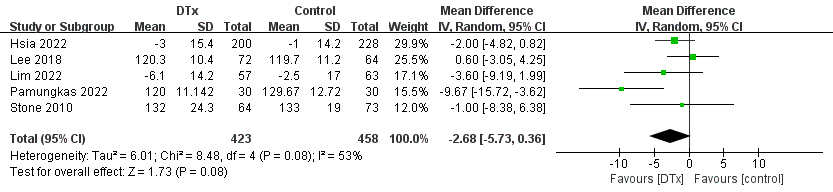


**Figure 8.** Forest plot showing effectiveness of DTx for diabetes management in improving DBP


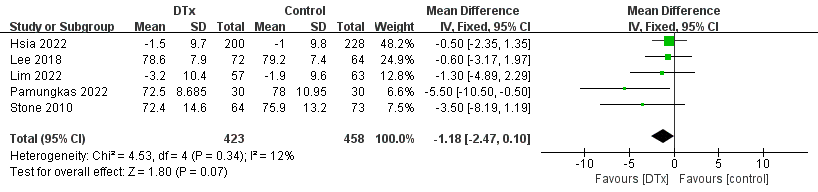


**Figure 9.** Forest plot showing effectiveness of DTx for diabetes management in improving total cholesterol


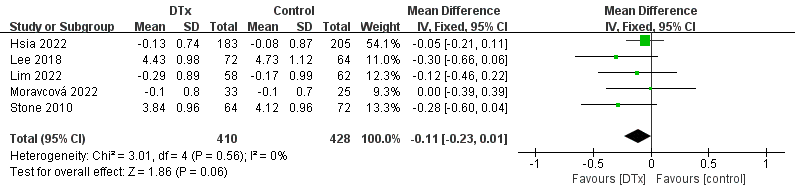


**Figure 10.** Forest plot showing effectiveness of DTx for diabetes management in improving HDL-C


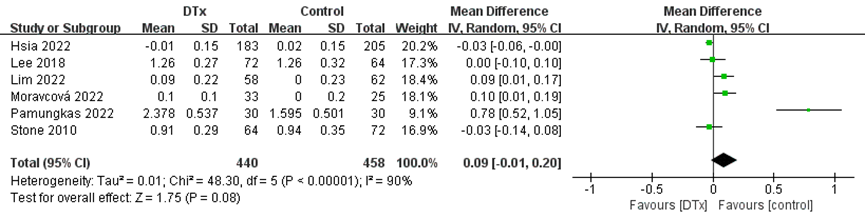


**Figure 11.** Forest plot showing effectiveness of DTx for diabetes management in improving LDL-C


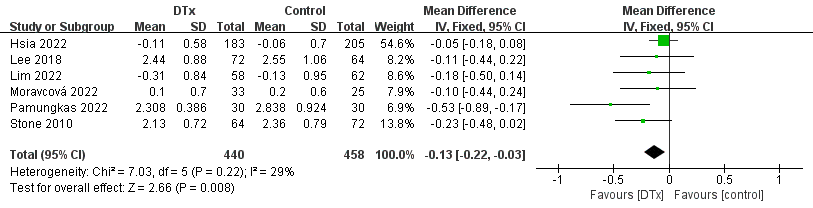


**Figure 12.** Forest plot showing effectiveness of DTx for diabetes management in improving triglycerides


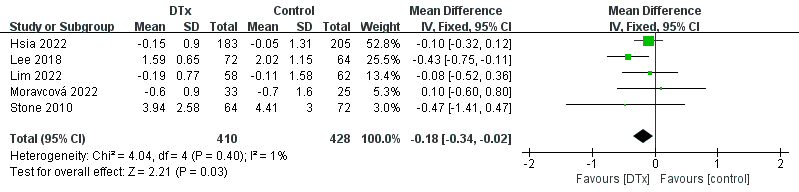


**Figure 13.** Baujat plot of HbA1c


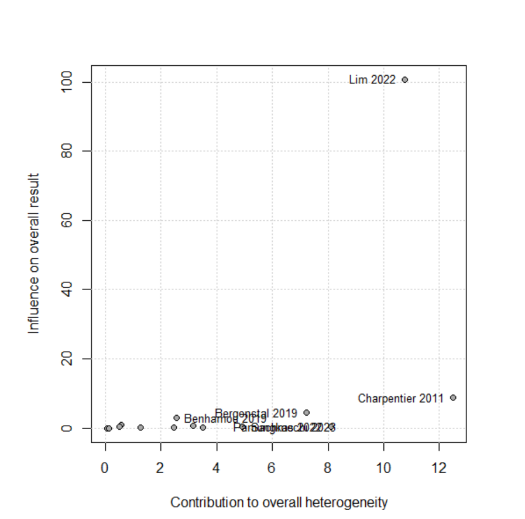


**Figure 14.** HbA1c Funnel plot

**
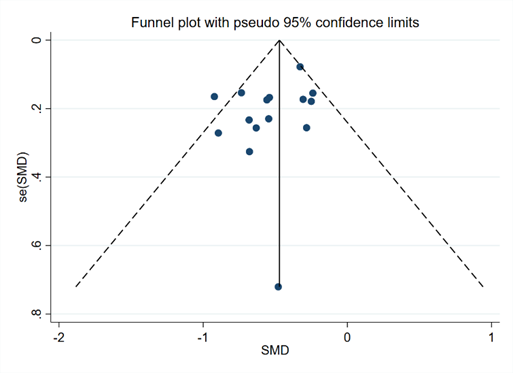
**

**Figure 15.** Forest plot for sensitivity analysis


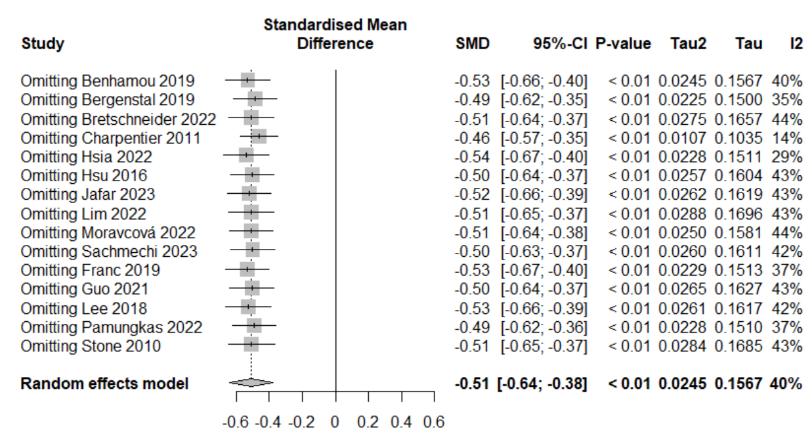

Supplement: Multimedia Appendix 8 [file jmir_v27i1e70428_app8.docx]
